# Visualization of lignification in flax stem cell walls with novel click-compatible monolignol analogs

**DOI:** 10.3389/fpls.2024.1423072

**Published:** 2024-08-19

**Authors:** Lan Yao, Rui Wang, Chang Geun Yoo, Yuhang Zhang, Xianzhi Meng, Wei Liu, Arthur J. Ragauskas, Haitao Yang

**Affiliations:** ^1^ Key Laboratory of Fermentation Engineering (Ministry of Education), Cooperative Innovation Center of Industrial Fermentation (Ministry of Education and Hubei Province), College of Bioengineering, Hubei University of Technology, Wuhan, China; ^2^ Hubei Provincial Key Laboratory of Green Materials for Light Industry, Hubei University of Technology, Wuhan, China; ^3^ Department of Chemical Engineering, State University of New York College of Environmental Science and Forestry, Syracuse, NY, United States; ^4^ State Key Laboratory of Biobased Material and Green Papermaking, Qilu University of Technology, Shandong Academy of Sciences, Jinan, Shandong, China; ^5^ Department of Chemical and Biomolecular Engineering, University of Tennessee Knoxville, Knoxville, TN, United States; ^6^ Joint Institute for Biological Sciences, Biosciences Division, Oak Ridge National Laboratory (ORNL), Oak Ridge, TN, United States; ^7^ Department of Forestry, Wildlife, and Fisheries, Center for Renewable Carbon, The University of Tennessee Knoxville, Institute of Agriculture, Knoxville, TN, United States

**Keywords:** lignin, click chemistry, monolignol analog, alkyne group, flax

## Abstract

**Introduction:**

As an essential part of plant cell walls, lignin provides mechanical support for plant growth, enhances water transport, and helps to defend against pathogens. As the most abundant natural aromatic-based renewable resource on earth, its biosynthesis has always been a research focus, and it is still currently under study.

**Methods:**

In this study, the *p*-coumaryl alcohol analog (H_ALK_) and the coniferyl alcohol analog (G_ALK_) containing an alkyne group at the ortho position were synthesized and applied to lignification *in vivo* and *in vitro*. The incorporation of these novel lignin monomers was observed *via* fluorescence imaging.

**Results and Discussion:**

It was found that the two monolignol analogs could be incorporated in dehydrogenated polymers (DHPs) *in vitro* and in flax cell walls *in vivo*. The results showed that as the cultivation time and precursor concentration varied, the deposition of H and G-type lignin exhibited differences in deposition mode. At the subcellular scale, the deposited lignin first appears in the cell corner and the middle lamella, and then gradually appears on the cell walls. Furthermore, lignin was also found in bast fiber. It was demonstrated that these new molecules could provide high-resolution localization of lignin during polymerization.

## 1 Introduction

With the development of science and technology, more and more chemicals containing aromatic units are being utilized, and these chemicals rely mainly on non-renewable petroleum resources. However, chemicals containing aromatic units are often difficult to synthesize commercially, and there are significant environmental concerns and possible resource shortages in the near future (Ragauskas et al., 2006; Calvo-Flores and Dobado, 2010). Hence, more research is now focused on the efficient application of renewable biomass resources (Sun et al., 2018). Lignin is the most abundant aromatic polymer on Earth. It is also one of the most important renewable biomass macromolecules composed of aromatic subunits in nature so far and is considered an ideal alternative to replace petroleum-based phenolic chemicals (Luterbacher et al., 2014).

Lignin is a complex and heterogeneous polymer, which is critical to secondary cell wall integrity (Vanholme et al., 2010). Lignification of plants in specialized cells can resist the attack of pathogens and herbivores (Boerjan et al., 2003; Bonawitz and Chapple, 2010). Lignin deposition is a dynamic and complex process in which monomers are first synthesized in the cytoplasm through the phenylalanine pathway (Chiang, 2006; Umezawa, 2009), followed by their transportation to the extracellular space, and are oxidized by peroxidase or laccase to produce free radicals. Finally, lignin can be polymerized through free radical coupling reactions (Ralph et al., 2004, 2008). Although tremendous progress has been made (Liu, 2012; Vanholme et al., 2013; Liu et al., 2018), the exact lignin deposition mechanisms are still not fully understood.

Recently, fluorescence imaging technology has gradually become a powerful tool for studying biological molecular processes, which could be applied to observe the subcellular localization of fluorescent labeling molecules (Watson et al., 2005; Lavis et al., 2006; McIntosh et al., 2008). Furthermore, the transportation and polymerization of lignin monomers can be visualized at the cell level. In recent studies, coniferyl alcohol model compounds with azide and alkynyl labeling were designed and synthesized. *Arabidopsis thaliana* tissues were cultured with the model compounds to study the deposition mode (Tobimatsu et al., 2014). Bukowski and Pandey designed and synthesized 3-O-propargylcaffeyl alcohol (3-OPC), which was incorporated in the tissue of *Arabidopsis thaliana* to study the biochemical process and plant lignification process (Bukowski et al., 2014; Pandey et al., 2016). Due to the absence of methoxyl groups in the modified monomer, the information on the synthesis and deposition of S-type and H-type lignin was limited in the whole observation process. Subsequently, a dual-labeling strategy was developed to detect the lignification process of two different model compounds (H type and G type) simultaneously (Simon et al., 2017). A small molecule azide group and alkyne group were introduced into the methoxy group of coniferyl alcohol and the γ position of *p*-coumaryl alcohol, respectively. They were cultivated with plants together, and then the lignification process was observed through a click chemical reaction (Lion et al., 2017). Due to the dual-labeling technology, interference from other components from the plant cell wall was reduced, making the results more reliable. However, the information on the synthesis and deposition of S-type and part of G-type and H-type lignin is still missing in the whole lignification process due to the absence of hydroxyl groups at the γ position and the methoxy group attached to the benzene ring.

Bioorthogonal reaction refers to a class of chemical reactions that can be carried out in living cells without interfering with their own biological processes (Sletten and Bertozzi, 2009). The application of bioorthogonal chemistry includes two processes: first, the incorporation of bioorthogonal reporter molecules into target biomolecules, and then the bioorthogonal reaction of the probe molecule. It has unique advantages, including wide application and high scalability (Lim and Lin, 2010). Due to the chemical polymerization mode of lignin, click model compounds to participate in the lignin deposition process could be applied to detect the lignification process of plant tissues effectively.

In this study, information groups were introduced at the sites where lignin monomers do not participate in the lignification process (such as the ortho position of the aromatic ring). Specifically, two new lignin analogs, 2-O-proparyl *p*-coumaryl alcohol (2-O-PPA or H_ALK_) and 2-O-proparyl coniferyl alcohol (2-O-PCA or G_ALK_), were designed and incorporated into flax *in vitro* to explore the deposition mode of lignin in the flax cell wall. Therefore, the mechanism of the lignification of plant cell walls can be fully revealed. The results could provide a theoretical basis for lignin deposition, and help to promote the efficient separation and valorization of lignin.

## 2 Materials and methods

### 2.1 Plant material

Flax plants were used for all experiments. For experiments on cross-sections, 2-month-old flax was grown from seeds sown in compost and grown outdoors in January 2023 in Wuhan, Hubei province, China.

### 2.2 Lignin monomers

The lignin precursors, namely, *p*-coumaryl alcohol (*p*CA), sinapyl alcohol (SA), coniferyl alcohol (CA), H_ALK_ and G_ALK_, were prepared by *p*-coumaric acid, sinapic acid, ferulic acid, *p*-hydroxybenzaldehyde, and vanillin, respectively. See the Supplemental Information for detailed synthesis protocols for *p*CA, SA, and CA (**Supplementary Figures S1**–**S7**); H_ALK_ (**Supplementary Figures S8**–**S13**); and G_ALK_ (**Supplementary Figures S14**–**S21**).

### 2.3 Biosynthesis of GSH lignin dehydrogenation polymers

G:S:H of 2:2:1 was prepared with different H labeling ratios, i.e., 80 mg of coniferyl alcohol, 80 mg of sinapyl alcohol, 40 mg of *p*-coumaryl alcohol; 80 mg of coniferyl alcohol, 80 mg of sinapyl alcohol, 30 mg of *p*-coumaryl alcohol, 10 mg of H_ALK_; 80 mg of coniferyl alcohol, 80 mg of sinapyl alcohol, 20 mg of *p*-coumaryl alcohol, 20 mg of H_ALK_; and 80 mg of coniferyl alcohol, 80 mg of sinapyl alcohol, 40 mg of H_ALK_. Lignin monomers were added in a 250 mL conical flask with 5 mL of acetone, 195 mL of PBS (0.01 M, pH=6.5), and 3 mg horseradish peroxidase (Sigma-Aldrich, 153.18 U/mg). Furthermore, 0.025% H_2_O_2_ solution and monomer solution were added dropwise through a constant flow pump to 2 mL PBS containing 1 mg horseradish peroxidase for about 67 hours at room temperature (3 mL/h). After stirring at room temperature for another 24 hours, the precipitate was obtained after centrifuging at 8000 rpm for 20 minutes at 4°C to remove the unreacted phosphate buffer and enzyme. The dehydrogenated polymer (DHP) was obtained after washing with deionized water four times and followed by freeze drying.

### 2.4 Biosynthesis of H lignin dehydrogenation polymers

A solution of *p*-coumaryl alcohol (or H_ALK_) was prepared in a pH 6.5 phosphate buffer solution. The synthesis method was the same as GSH-DHP.

### 2.5 Nuclear magnetic resonance spectroscopy determination

Initially, 50 mg DHP was dissolved in 0.5 mL DMSO-*d*_6_. A Bruker Avance 600 MHz spectrometer equipped with a 5-mm Broadband Observe probe was used to perform 2D-HSQC NMR. A Bruker standard pulse sequence was used for analysis, with a spectral width of 11 ppm in F2 (^1^H) of 2048 data points and 190 ppm in F1 (^13^C) of 256 data points. The scanning delay of 1-s and 56 scans were performed. MestReNova was applied for NMR spectra analysis.

### 2.6 Click labeling of DHPs and fluorescence measurements

Ten milligrams of freeze-dried H-DHP, 25% H(H_ALK_)-DHP, 100% H_ALK_-DHP, GSH-DHP, 25% GSH(H_ALK_)-DHP, 50% GSH(H_ALK_)-DHP, or 100% GSH_ALK_-DHP were dissolved in 2 mL click labeling solution (1 mM L-ascorbic acid, 1 mM CuSO_4_, and 1 μM azide fluor-545 solution in Murashige and Skoog culture medium). The solution was kept at 25°C for 1 h in a shaker. The DHP suspension was obtained by centrifugation (8000 r/min, 4°C, 20 min) and washed with distilled water four times (8000 r/min, 4°C, 20 min) to remove monomers that do not react with fluorescent groups. The final product was obtained after freeze drying.

Unlabeled H-DHP, GSH-DHP, labeled H-DHP, GSH-DHP, 25% H(H_ALK_)-DHP, 50% H(H_ALK_)-DHP, 100% H_ALK_-DHP, 25% GSH(H_ALK_)-DHP, 50% GSH(H_ALK_)-DHP, and 100% GSH_ALK_-DHP solutions (5 mg/mL) were prepared in dimethyl sulfoxide (DMSO). An F-7000 fluorescence spectrophotometer (Hitachi Koki Co., Ltd. Tokyo, Japan) was used for analysis. The band-pass filter was set to 5 nm, and the spectral integration time was 0.1 s. λ_ex_ of 561 nm and λ_em_ of 545-750 nm were applied.

### 2.7 FT-IR spectroscopy determination

First, 0.5 mg oven-dried DHP and 20 mg potassium bromide (KBr) were ground into fine powder and made into pellets. FT-IR spectroscopy (Nicolet 6700, Thermofisher Nicolet, America) was conducted on the samples with 64 scans from 4000 cm^-1^ to 500 cm^-1^, with 2 cm^-1^ resolution. The spectra were observed and processed by OMNIC software.

### 2.8 UV spectroscopy determination

Lignin dehydrogenated polymer (5 mg) was dissolved in 10 mL of 95% (V/V) dioxane solution, which was diluted to 100 mL with 50% (V/V) dioxane solution. A UV spectrophotometer (UV-2550, Shimadzu Co., Ltd., Japan) was employed to scan the solution at the wavelength of 200 nm~450 nm, with a 2 nm slit width and 0 s delay with 600 nm/min scan speed. Dioxane solution (50%) was used as a control. Origin was applied for UV spectra analysis.

### 2.9 Incorporation and labeling of flax stem sections

Two-month-old flax was cultivated with vertical support. The stem of the flax was cut horizontally 10 cm above the soil level and was then cut into several cross sections (about 150-250 µm thick) (Simon et al., 2018). To cultivate flax with different times (time group), 100 µM G_ALK_/H_ALK_/*p*-coumaryl alcohol/coniferyl alcohol was dissolved in 300 µL sterile Murashige and Skoog culture medium (2.2 g MS basic medium with 0.6 g 4-morpholineethanesulfonic acid, pH 5.6), respectively. H_ALK_ and *p*-coumaryl alcohol and G_ALK_ and coniferyl alcohol (each 10 µM), prepared with Murashige and Skoog culture medium, were incubated at 20°C for 20 h. After incorporation, the sections were washed with 500 µL of liquid MS medium four times. Each solution was then transferred to a click labeling solution (2.5 mM L-ascorbic acid, 0.5 mM CuSO_4_, and 5 µM azide-fluor 545), which was kept in the dark for 1 h. After being washed twice with 1 mL Murashige and Skoog culture medium, sections were then transferred to 70% MeOH for 1 h to remove any unbound monomers or dyes and finally washed with 1 mL Murashige and Skoog culture medium four times. In the group of chemical reporters with different concentrations, the flax sections were treated with 1 mM lignin precursors, which contained 0%, 1%, 10%, 25%, 50%, 75%, or 100% G_ALK_ or H_ALK_ for 20 h, and click labeling was conducted with CuAAC (25 mM L-Ascorbic acid, 5 mM CuSO_4_, 50 μM Azide-fluor 545) for 1 h. The following procedure was the same as the time group.

### 2.10 Image acquisition by confocal laser scanning microscope

After washing, stem sections were set in a fluorescent mounting medium, and stored in the dark at 4°C. A confocal laser scanning microscope, a Leica SP8, using 20× and 63× (oil immersion) objectives was used for image acquisition. The Lignin spontaneous fluorescence channel was set as λ_ex_ 405 nm and λ_em_ 450 nm. 5-Carboxytetramethylrhodamine azide (azide-fluor 545 or AF545) dye fluorescence(CuAAC) channel was set as λ_ex_ 552 nm and λ_em_ 595 nm. ImageJ was applied to generate the maximum projection of the z series. After selecting lignified regions using a common threshold, the fluorescence intensity was quantified as the original integral intensity per unit area in ImageJ. Separate thresholds were set for images acquired under 405 nm and 552 nm channels. There were 10 replicates in each group, the fluorescence intensity values were expressed as mean ± SD, and images were collected three times for each sample. Statistical analysis was performed by SPSS.

## 3 Results

### 3.1 The characteristics of DHPs

With the help of H_2_O_2_ and horseradish peroxidase, the yields of H-DHP (yield of 47.5%), 15% H(H_ALK_)-DHP (yield of 45%), 25% H(H_ALK_)-DHP (yield of 50%), 50% H(H_ALK_)-DHP (yield of 67%), and 100% H_ALK_ -DHP (yield of 57%) were obtained. GSH-DHP yielded between 51%-64%.

#### 3.1.1 FT-IR analysis

**Figure 1:**
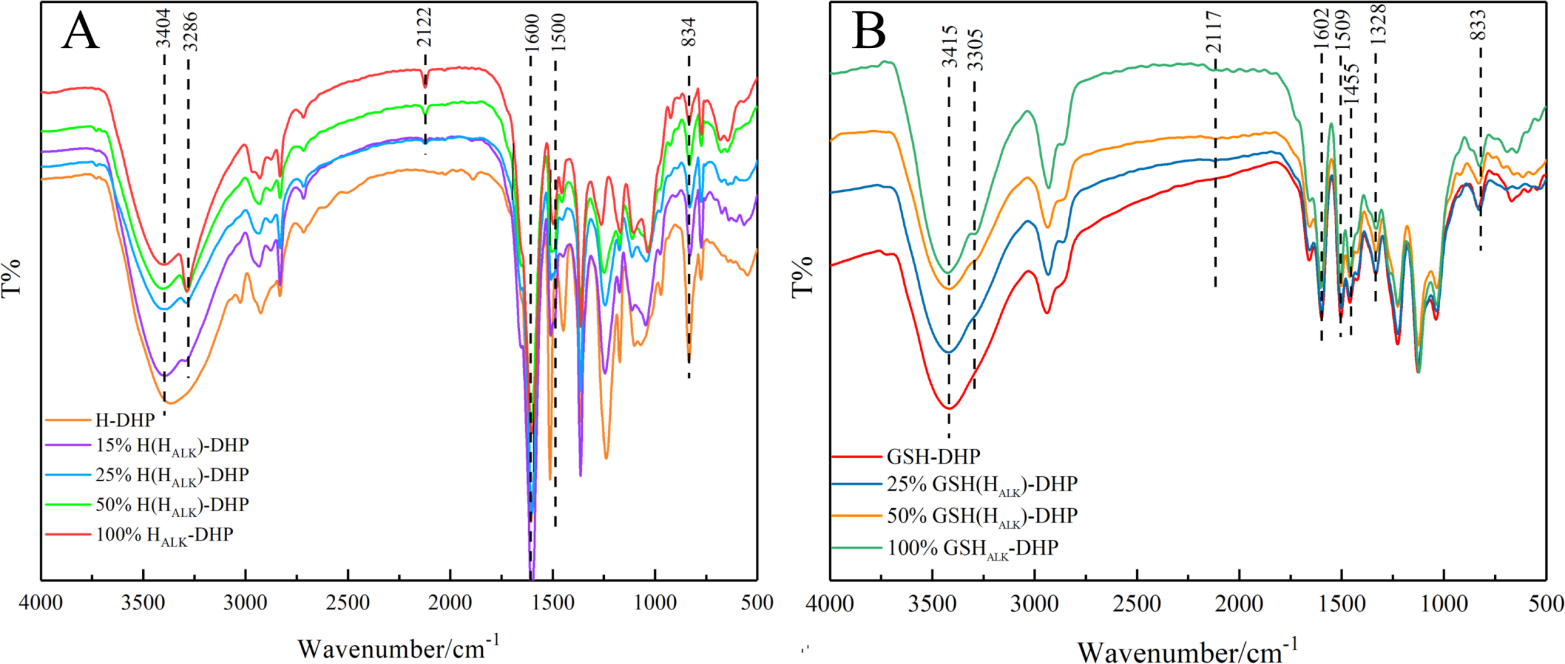
FTIR spectra of DHPs. **(A)** H-DHP; **(B)** GSH-DHP.

FT-IR is widely applied to analyze the structure of lignin (Halina et al., 2020). The structure of lignin can be studied based on FT-IR spectra to determine various functional groups and chemical bonds, such as carbonyl, hydroxyl, methoxy, C-H, and C-C linkages (Liu et al., 2014). The FT-IR spectra of DHPs are shown in **Figure 1**. There was a strong and wide signal representing hydroxyl (-OH) around 3404 cm^-1^, which was mainly due to aliphatic and phenolic hydroxyl groups. Peaks at 3286 cm^-1^ and 2122 cm^-1^ were from C≡CH and C≡C, respectively, indicating that H_ALK_-DHP contained propargyl groups. There were obvious signals from the benzene ring at wavenumbers of 1600 cm^-1^, 1500 cm^-1^, and 1455 cm^-1^, which proved that the structure of synthesized DHP was close to lignin. The signal at 834 cm^-1^ in H-DHP represents the C-H vibration of *p*-hydroxyphenyl aromatic ring. Furthermore, wavebands at 1268 cm^-1^ and 1140 cm^-1^, which were assigned to the C-O absorption of the methoxyl groups in guaiacyl lignin and the C-H absorption of guaiacyl aromatic ring, were clearly observed in GSH-DHP. A C-H absorption peak from the *p*-hydroxyphenyl aromatic ring at 833 cm^-1^ was also found. In addition, the methoxy absorption peak of the syringyl ring at 1328 cm^-1^ in GSH-DHP indicated the structure of GSH lignin (Karmanov and Derkacheva, 2013; Lou et al., 2013).

#### 3.1.2 UV analysis

**Figure 2:**
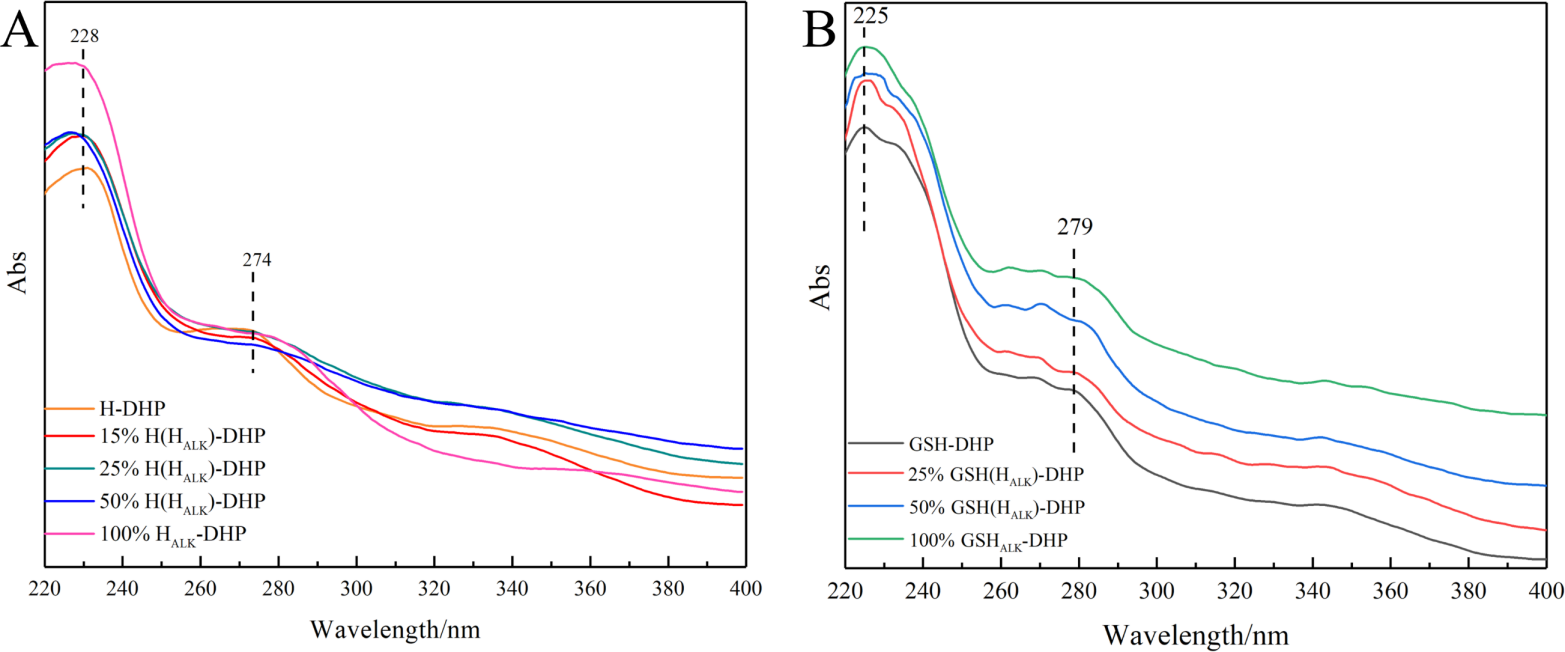
UV spectra of DHPs. **(A)** H-DHP; **(B)** GSH-DHP.

Lignin is a typical aromatic compound that shows a UV characteristic absorption between 250 nm - 400 nm (Sadeghifar and Ragauskas, 2020; Zhang and Naebe, 2021). It was clear that the signals at 274 nm from the benzene ring and 228 nm from the C=C bond conjugated with the aromatic ring in the UV spectra of H-DHP (**Figure 2**). Similarly, the UV spectra of GSH-DHP showed absorption at 279 nm, assigned to the benzene ring, and at 225 nm for the C=C bond conjugated to the aromatic ring. The various wavelengths assigned to the benzene ring for different DHPs were mainly due to their different number of methoxy groups. Therefore, the UV spectra showed that the DHPs contained the benzene ring and C=C bond on the side chain.

#### 3.1.3 2D-HSQC NMR analysis of DHP

**Figure 3:**
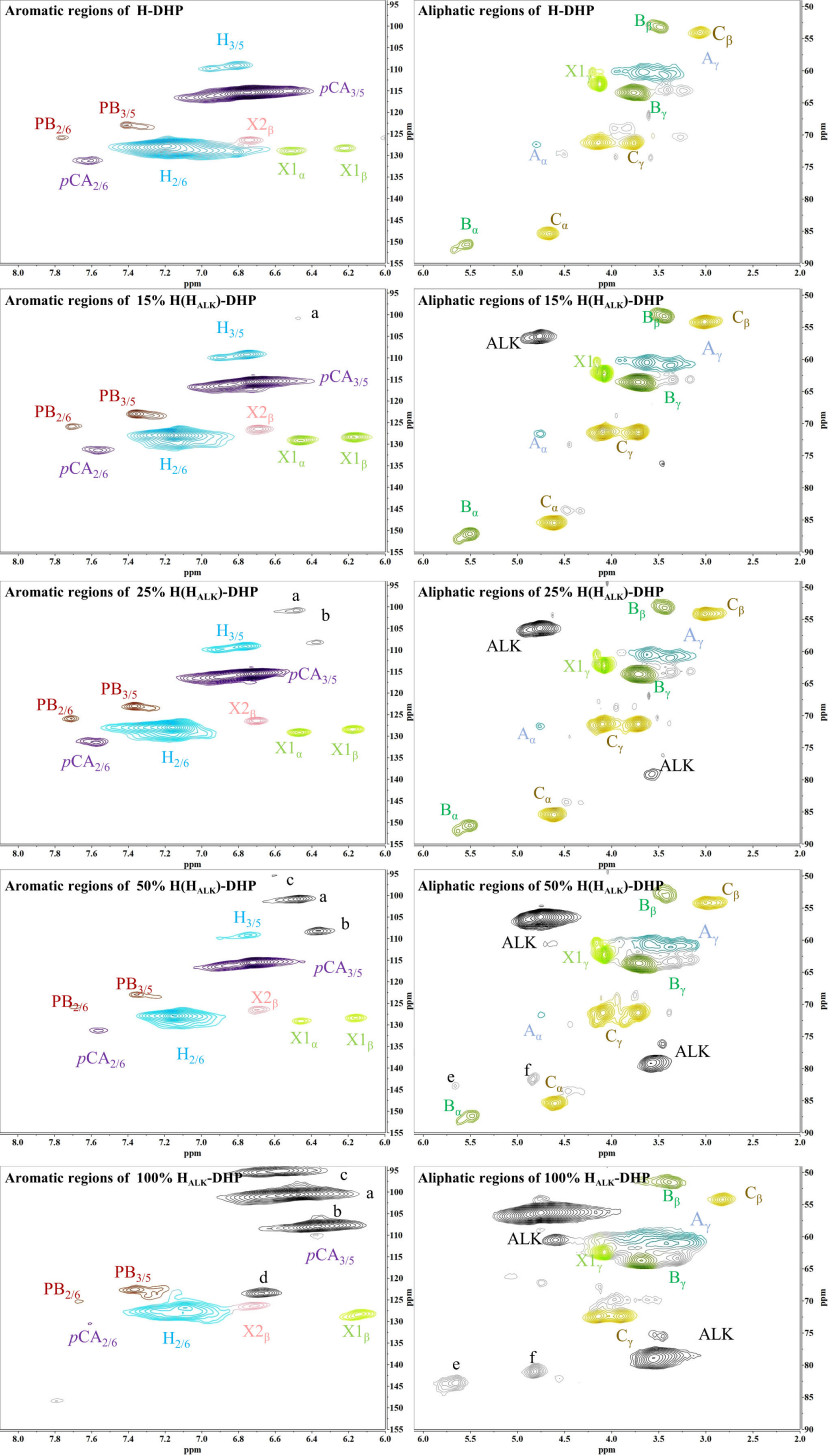
2D-HSQC NMR spectra of H-DHP. (A) New shift. a, b, c, d, e, f.

**Figure 4:**
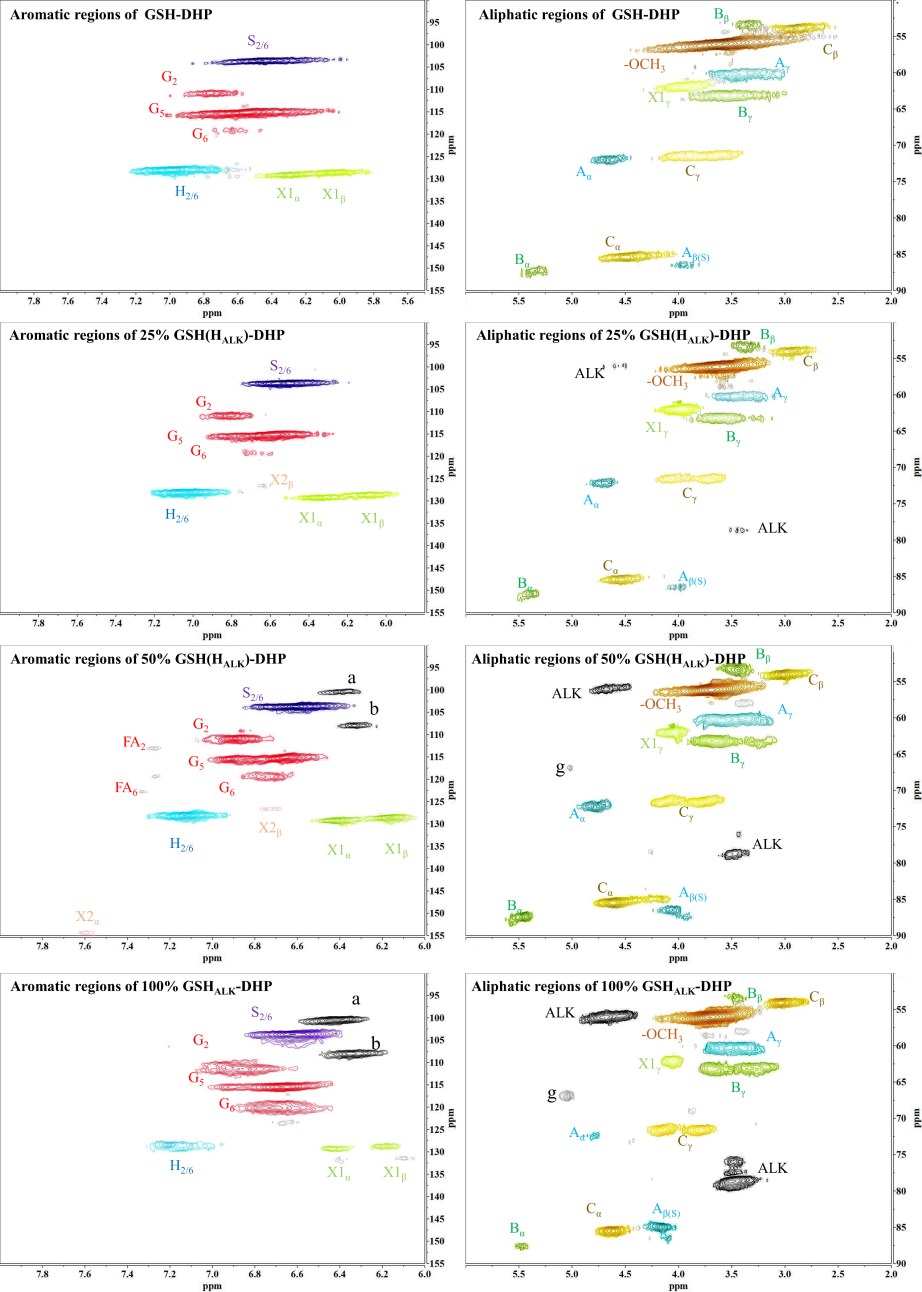
2D-HSQC NMR spectra of GSH-DHP. (A) New shift. g.

In order to assess whether the lignin precursors, H_ALK_, could participate in free radical coupling in lignin polymerized *in vitro*, different ratios of DHPs containing H_ALK_, coniferyl alcohol, sinapyl alcohol and *p*-coumaryl alcohol were prepared, and their 2D-HSQC NMR spectra are shown in **Figures 3** and **4**.

**Figure 5:**
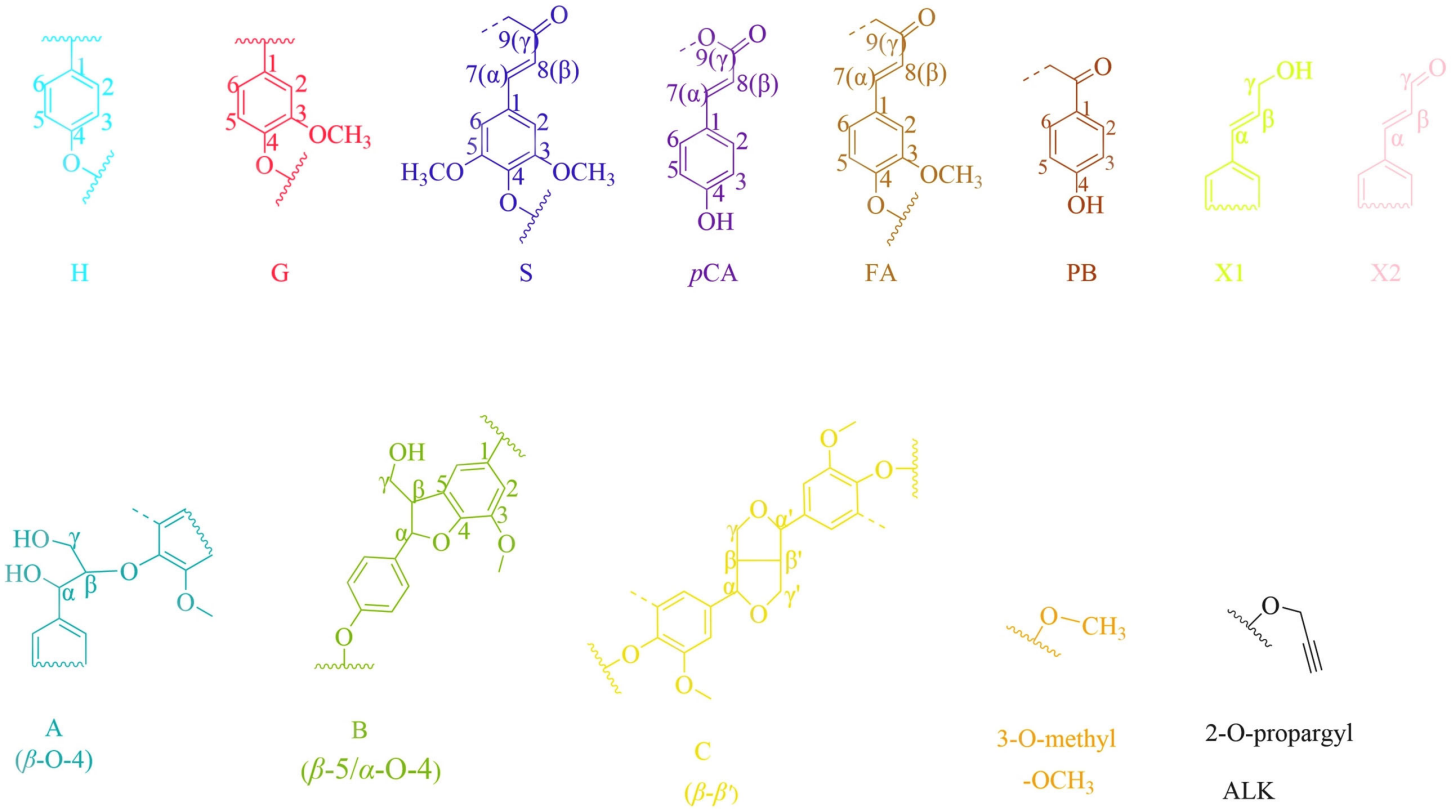
Important lignin substructure in 2D-HSQC NMR spectra.

Important substructures were shown in **Figure 5**. The aromatic region of DHP mainly included cross-peaks from H, G, *p*CA (*p*-coumaric acid), S (sinapyl alcohol), FA (ferulic acid), X1 (cinnamyl alcohol), X2 (cinnamaldehyde), and PB (*p*-hydroxybenzoate). Signals assigned to the *β*-O-4 alkyl ether bond (A), *β*-5/*α*-O-4 (B), *β-β’* structure (C), methoxy group, and two C-H signals corresponding to the alkyne side chain on H_ALK_ were detected in the aliphatic region (Bukowski et al., 2014; Guo et al., 2015; Obame et al., 2019; Wang et al., 2019).

When the content of H_ALK_ was increased, new signals at 100.60/6.47 ppm (a), 107.93/6.37 ppm b), 95.14/6.57 ppm (c), 123.43/6.67 ppm (d), 82.80/5.65 ppm (e), 81.05/4.81 ppm (f), and 66.88/5.04 ppm (g) were observed. The intensity of signals from a, b, c, e, f, g, Alk1 (56.25/4.75 ppm) (Kim and Ralph, 2010). and Alk2 (78.94/3.55 ppm) was enhanced with the increased content of H_ALK_. In addition, when the content of H_ALK_ was increased, the signal intensity of A_α_ (71.58/4.80 ppm) was decreased. New chemical shifts of e (82.73/5.63 ppm) and f (81.71/4.83 ppm) could be found in 50% H(H_ALK_)-DHP, and the highest peak intensity was with 100% H_ALK_-DHP. However, the two signals were low in 25% H(H_ALK_)-DHP. Due to the low content of alkyne groups, fewer effects on B_α_ of *β*-5 and C_α_ of *β-β* were observed. The alkyne content in 100% GSH_ALK_-DHP was the same as 20% H(H_ALK_)-DHP, and chemical shifts of e and f disappeared.

#### 3.1.4 Fluorescence spectra analysis of DHPs

**Figure 6:**
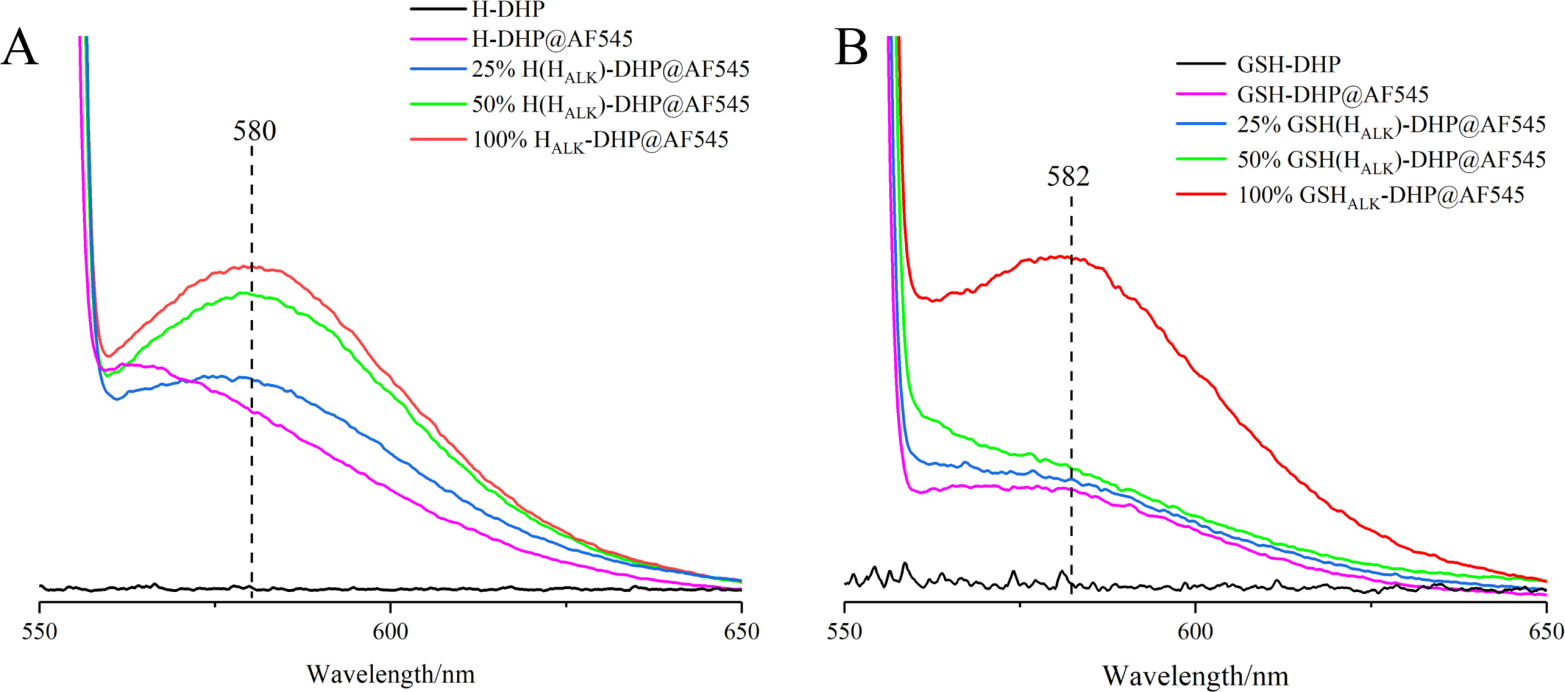
Fluorescence spectra of DHPs. **(A)**, H-DHP; **(B)**, GSH-DHP.

The prepared DHP was fluorescent-labeled after the click reaction. The fluorescence spectra showed that DHPs containing H_ALK_ exhibited higher fluorescence intensity (25% H_ALK_-DHP@AF545 is 3.95 a.u.) than DHP without labeling (H-DHP is 0.26 a.u.) under the same click labeling conditions (**Figure 6**). With the enhanced content of H_ALK_, fluorescence intensity was increased from 3.95 a.u. (25% H_ALK_-DHP@AF545) to 5.96 a.u. (100% H_ALK_-DHP@AF545). GSH-DHP followed the same rule. Overall, the fluorescence intensity of GSH-DHP was weaker than that of H-DHP due to the lower amount of H_ALK_ in GSH-DHP. DHP without click labeling was applied as the negative control, having negligible fluorescence at 545 nm. The labeled DHPs showed a small amount of fluorescence at 580 nm, but it was much lower than the DHP containing H_ALK_.

### 3.2 Incorporation of H_ALK_ and G_ALK_ in flax stem sections

**Figure 7:**
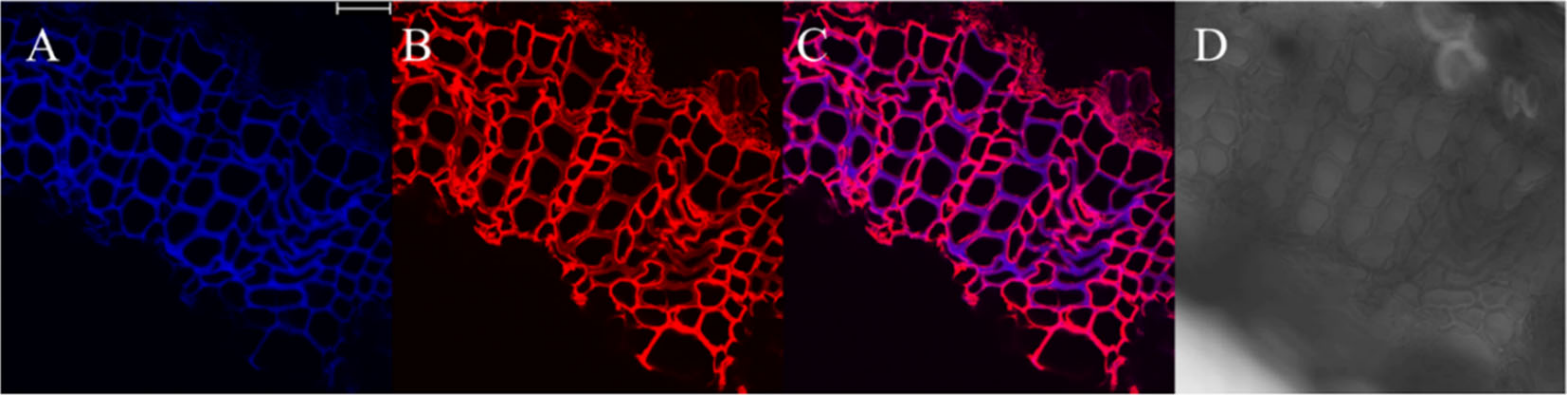
Image of cultivated flax stem sections under microscope (×63). **(A)** Lignin autofluorescence (405 nm, blue fluorescence). **(B)** Fluorescence after G_ALK_ incorporation (552 nm, red fluorescence). **(C)** Merged image of A and B **(D)** Imaging of the Flax stem sections in bright field. Scale bar, 25 μm.

**Figure 8:**
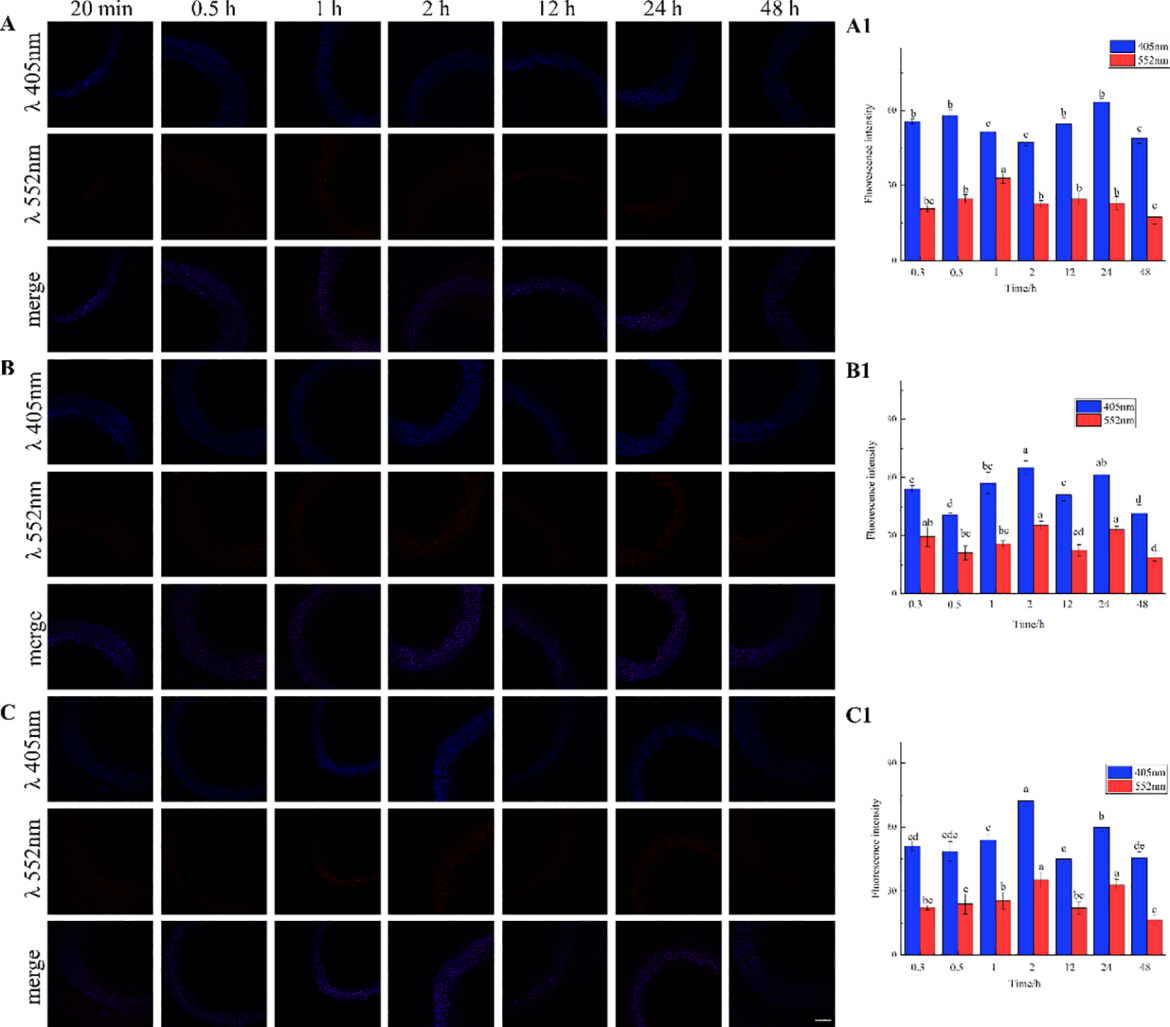
Imaging and Fluorescence intensity changes of flax stem sections cultivated with lignin precursors over time (×20). (**A**, A1) no lignin precursors; (**B**, B1) coniferyl alcohol; (**C**, C1) *p*-coumary alcohol. Scale bar, 50 μm. (CuAAC contains 2.5 mM L-Ascorbic acid, 0.5 mM CuSO_4_, 5 μM Azide fluor 545 dissolved in 1mL MS solution). Lignin autofluorescene (405nm), alkynyl labeled lignin fluorescene (552 nm). Values are expressed as mean of the fluorescence intensity ± SD.

**Figure 9:**
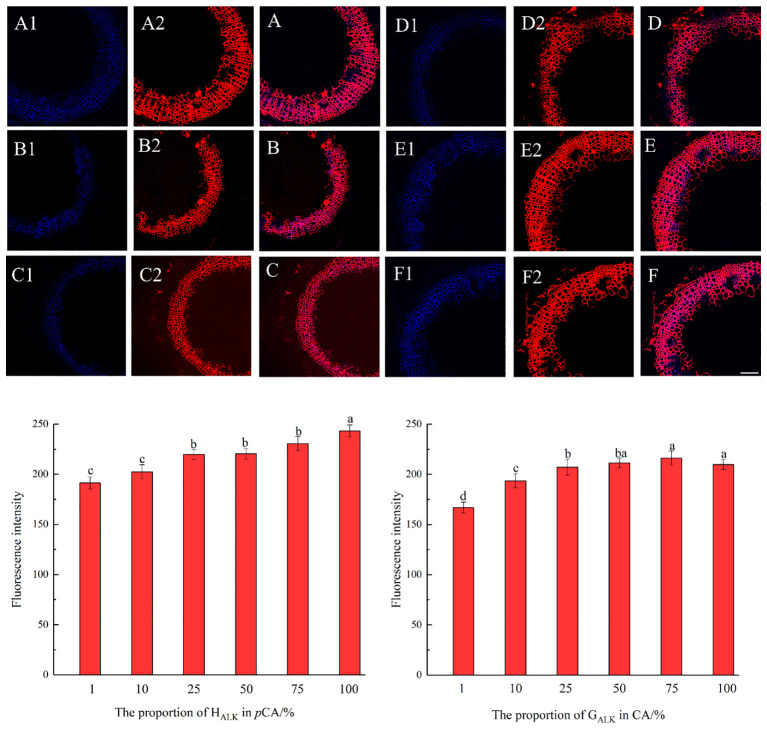
Imaging and Fluorescence intensity changes of flax stem sections cultivated with synthesized lignin at different concentrations (×20). **(A-C)** merged autofluorescence (blue) channel and clickable fluorescence (red) channel of 10%, 75%, 100% G_ALK_. **(D-F)** merged autofluorescence (blue) channel and clickable fluorescence (red) channel of 10%, 25%, 75% H_ALK_. **(A1-F1)** Autofluorescence of lignin at 405 nm. **(A2-F2)** Fluorescence of synthesized lignin at 552 nm. Scale bar, 50 μm. (CuAAC contains 25 mM ascorbic acid, 5 mM CuSO_4_, 50 μM Azide fluor 545 dissolved in 1mL MS solution).

**Figure 10:**
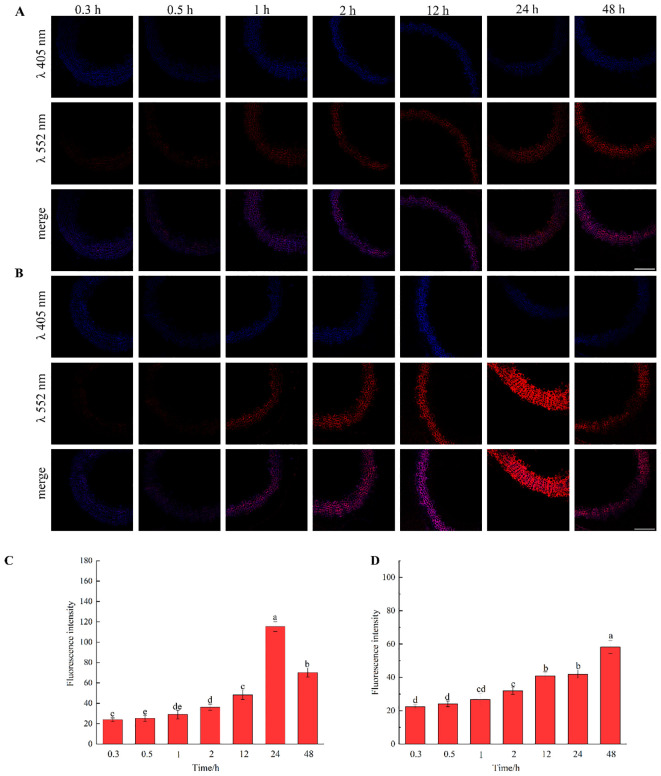
Imaging and Fluorescence intensity changes of flax stem sections cultivated with lignin precursors labeled with alkynyl structures over time (×20). **(A, C)** G_ALK_; **(B, D)** H_ALK_. Scale bar, 50 μm. (CuAAC contains 2.5 mM L-Ascorbic acid, 0.5 mM CuSO_4_, 5 μM Azide fluor 545 dissolved in 1mL MS solution).

The results, as shown in **Figure 7**, showed that the designed and synthesized G_ALK_ and H_ALK_ can be incorporated into plant cells and show fluorescence after click labeling with azide-fluor 545. At the excitation wavelength of 405 nm, the autofluorescence of lignin was observed (**Figure 7A**), while at 552 nm, cells labeled with azide-fluor 545 showed red fluorescence (**Figure 7B**), and the distribution could be seen in the bright field without fluorescence (**Figure 7D**). First, flax was treated with coniferyl alcohol and *p*-coumaryl alcohol for 0.3 h, 0.5 h, 1 h, 2 h, 12 h, 24 h, or 48 h, respectively. After labeling with CuAAC (2.5 mM L-ascorbic acid, 0.5 mM CuSO_4_, and 5 μM azide-fluor 545) for 1 h, the image of flax stem could be observed and quantified (**Figure 8**). The results showed that the two-month-old flax seedlings exhibited autofluorescence intensity of 50-60 a.u., and the red fluorescence intensity remained almost unchanged (20-30 a.u.) with increasing cultivation time. Therefore, the fluorescence intensity of intrinsic lignin remained almost unchanged, indicating that the flax used in the present study were all at the same growth period. Then, the flax section was treated with 1 mM lignin precursors, which contained 0%, 1%, 10%, 25%, 50%, 75%, or 100% G_ALK_/H_ALK_ for 20 h, and labeled with CuAAC. As shown in **Figure 9**, the fluorescence from click labeling could be observed in the middle lamella and cell walls. With the enhanced concentration of chemical reporters, the fluorescence intensity of stems incorporated with H_ALK_ increased gradually, while for G_ALK_, it increased sharply and was then stable when the concentration was over 50%. At the same time, 2-month-old flax was incubated in 100 μM G_ALK_ or H_ALK_ for 0.3 h, 0.5 h, 1 h, 2 h, 12 h, 24 h, or 48 h, respectively, and then labeled with CuAAC (**Figures 10A, B**). With the increase in cultivation time, the fluorescence intensity of stems incorporated with H_ALK_ was increased from 22.46 a.u. (0.3 h) to 58.20 a.u. (48 h). While for G_ALK_, the intensity rose from 23.99 a.u. (0.3 h) to 115.42 a.u. (24 h), then decreased to 70.26 a.u (48 h).

### 3.3 Lignin deposition in plant cells

**Figure 11:**
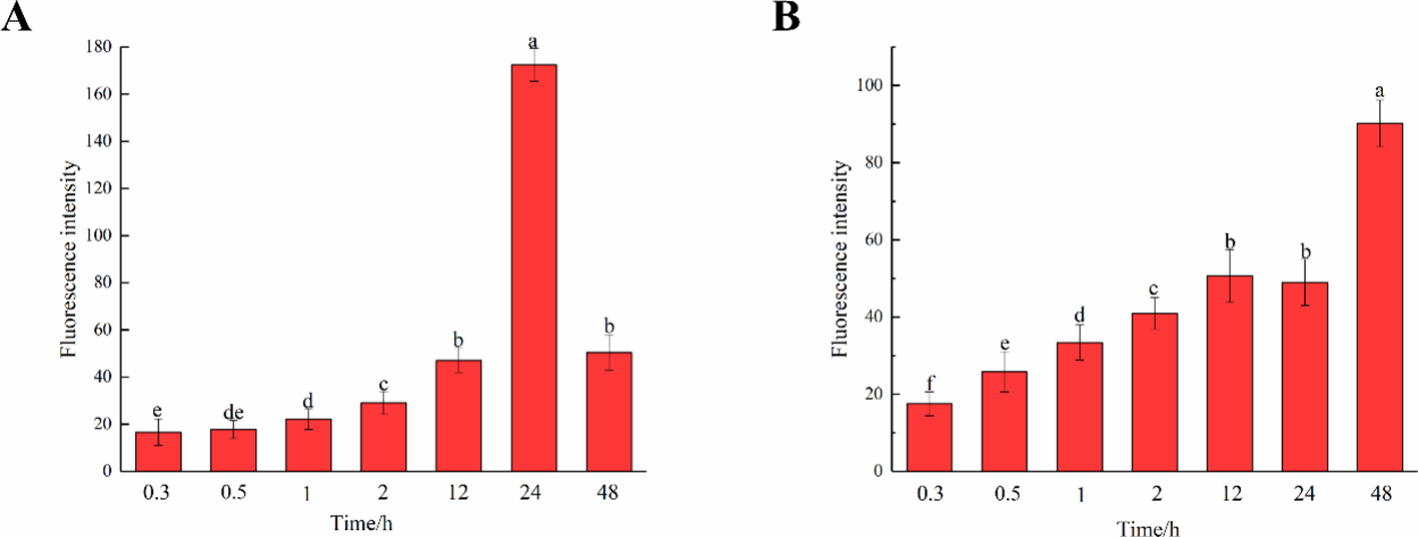
Fluorescence intensity of middle lamella over time. **(A)** G_ALK_. **(B)** H_ALK_. Values are expressed as mean of the fluorescence intensity ± SD. Different letters indicate statistical significance at p < 0.05.

The fluorescence intensity of cells in middle lamella at different times was observed and compared (**Figure 11**). As time progressed, the incorporation of both G and H lignin gradually increased. As shown in **Figures 10C, D**, the fluorescence intensity of the middle lamella was greater than that of the entire cell region, indicating that lignin was gradually and mainly deposited in the middle lamella of flax. The fluorescence intensity of the middle lamella cultured in H_ALK_ increased from 17.50 a.u. (0.3 h) to 90.20 a.u. (48 h), which was higher than the overall fluorescence intensity of stem cells [22.46 a.u. (0.3 h) to 58.20 a.u. (48 h)]. Meanwhile, the fluorescence intensity of the middle lamella cultured in G_ALK_ increased from 16.60 a.u. (0.3 h) to 172.40 a.u. (24 h), and then decreased to 50.40 a.u. at 48h, showing the same trend as the overall fluorescence intensity of stem cells. Overall, the fluorescence intensity of the middle lamella was higher than that of stem cells at 24 h (115.42 a.u.). Comparing the two bar charts in **Figure 11**, the two chemical reporters showed different trends of fluorescence intensity, which was in line with the results shown in **Figure 10**.

### 3.4 Cell specific differences in the xylem of flax

**Figure 12:**
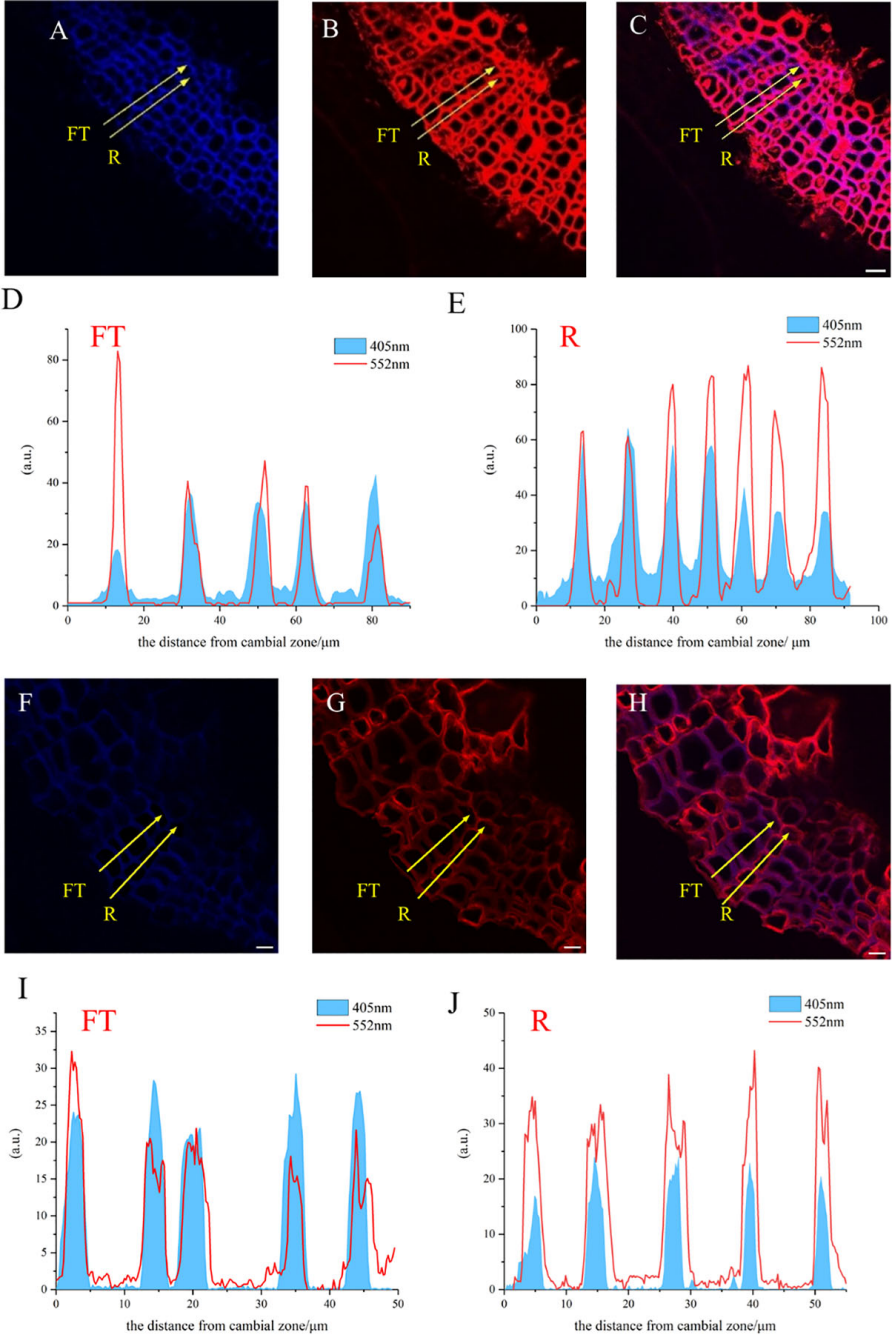
Lignification dynamics process in different cells. **(A-E)** G_ALK_. **(F-J)** H_ALK_. **(A, F)** Lignin autofluorescence (405 nm) in 24 h. **(B, G)** Fluorescence (552 nm) of synthesized lignin in 24 h. **(C, H)** Merged lignin autofluorescence and clickable fluorescence. **(D, I)** FT fluorescence in 405 nm and 552 nm channel, **(E, J)** R fluorescence in 405 nm and 552 nm channel, FT, Fiber tracheids, R, Ray parenchyma. The yellow arrow indicates the detection area of lignin fluorescence intensity. Scale bar,10μm.

A large portion of lignin exists in the secondary wall and middle lamella, which are mainly composed of relatively small diameter fiber tracheid tissue (FT) in flax and ray parenchyma cells (R). The results of the laser confocal microscope image confirmed the deposition of H_ALK_ (**Figure 12G**) and G_ALK_ (**Figure 12B**) into the cell wall of flax, including ray parenchyma, tracheid, and vascular. At the same time, a small amount of fluorescence can be observed in the bast fibers and parenchyma cells, indicating that there was also a small amount of lignin in these tissues for lignification.

The fluorescence intensity of ray parenchyma and fiber tracheid in 24 h was investigated and shown in **Figure 12**. After analyzing the fluorescence intensity of FT and R cells in flax under 552 nm, the fluorescence intensity of FT gradually decreased when the distance from the cambial zone increased (**Figure 12D**), while that of R increased (**Figure 12E**). The autofluorescence of R observed at 405 nm decreased (**Figure 12E**), while that of FT gradually increased (**Figure 12D**).

In the area closer to the cambium, the click labeling fluorescence intensity of FT was relatively high, while that of R was higher in part near the pith (**Figures 12D, E, I, J**). The 405 nm autofluorescence of FT near the cambium was lower than that of R, indicating that the closer to the cambium, the lower the degree of lignification of FT compared to R. In contrast, 405 nm autofluorescence of FT near the pith was higher than that of R (**Figures 12D, E, I, J**), which suggested a higher degree of lignification in FT than R in parts closer to the pith. Similar results were also observed in the H_ALK_-labeled flax (**Figures 12F-J**).

### 3.5 Reporters incorporated to reveal lignification process in single cell

**Figure 13:**
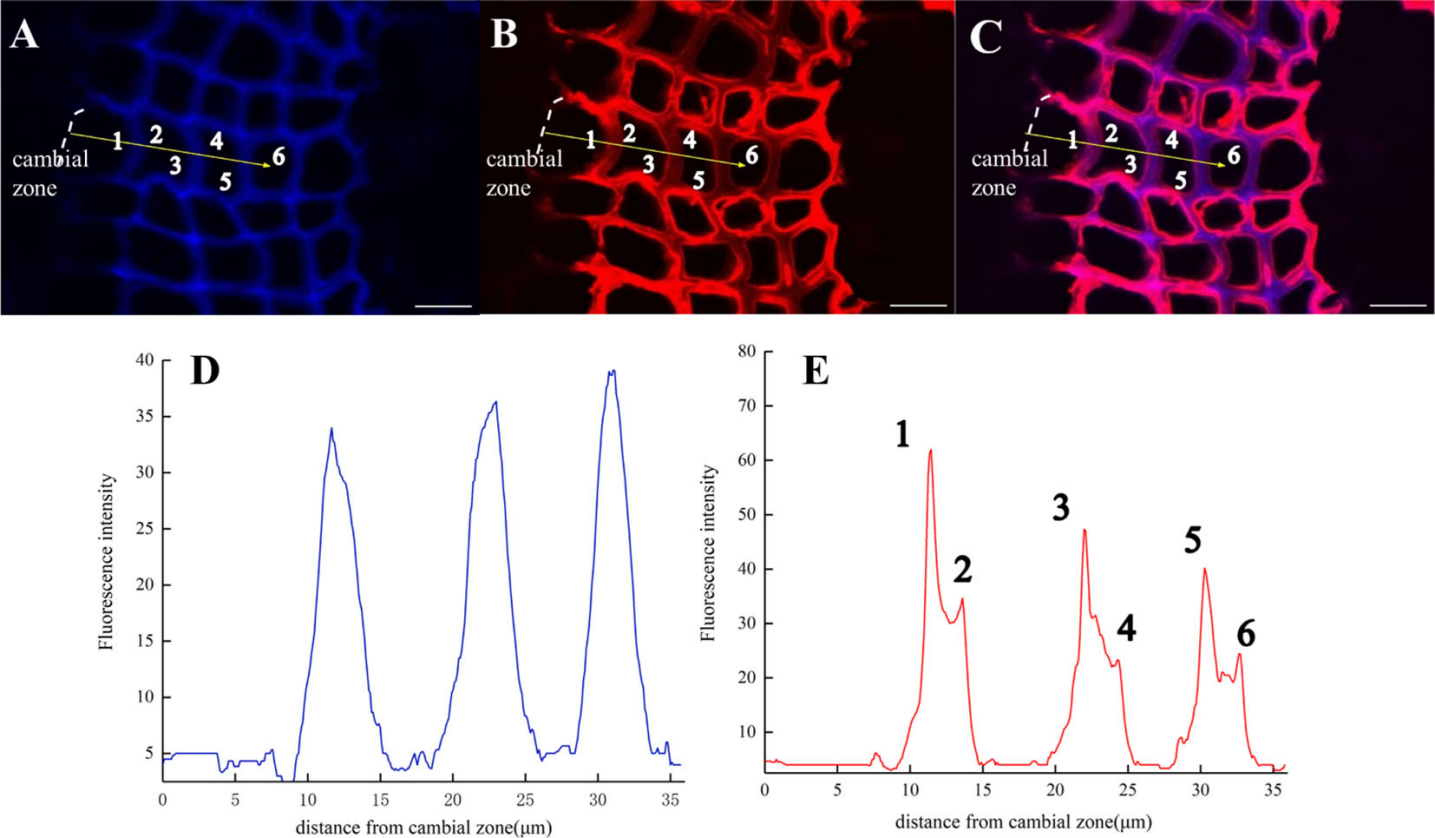
Lignification dynamics of Fiber tracheids (×63). **(A)** Lignin autofluorescence (405 nm) in 24 h. **(B)** Fluorescence (552 nm) of synthesized lignin in 24 h. **(C)** Merged lignin autofluorescence and G_ALK_ fluorescence. **(D, E)** Fluorescence intensity changes in the area with yellow arrows in **A, B**. Scale bar,10 μm.

After imaging analysis of the fiber tracheid (**Figure 13**), the results indicated that lignin precursors were first rapidly incorporated by cells in the middle lamella and then into S1 and S2, while the content of natural lignin rapidly increased in the middle lamella (**Figure 13D**). The peak width represents lignin accumulation in adjacent cell walls and the deposition of lignin in the middle lamella. The fluorescence intensity from artificially synthesized lignin was the highest in the first fiber tracheid, and it then gradually decreased in subsequent cell layers (**Figure 13E**). At the same time, it was also found that the fluorescence intensity of the cell wall [**Figure 13E** (1, 3, 5)] was significantly lower than that of the middle lamella at 24 h (**Figure 11**).

**Figure 14:**
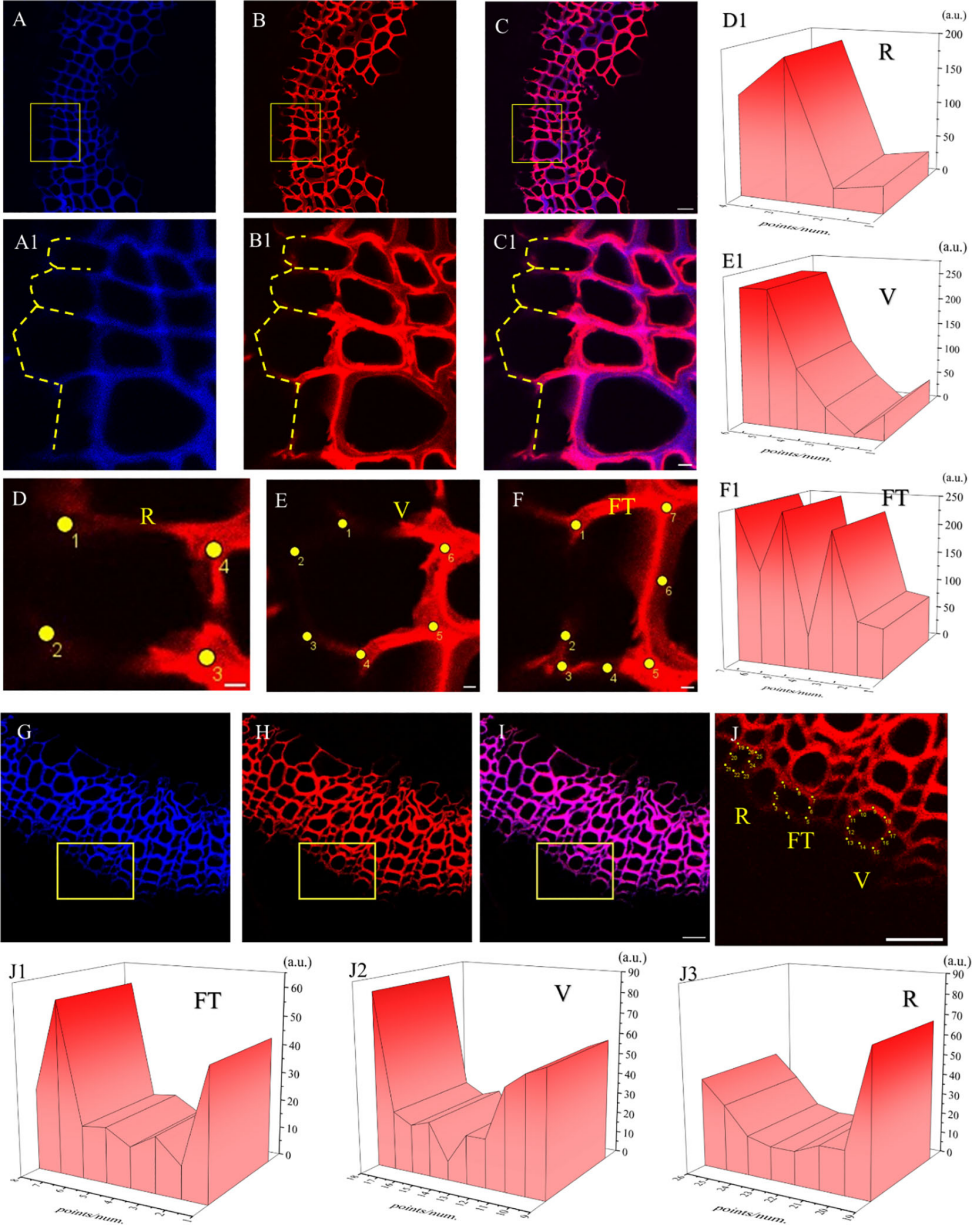
Imaging of Differentiating Cells (×63). **(A, G)** Lignin autofluorescence (405 nm). **(B, H)** Fluorescence (552 nm). **(C, I)** Lignin autofluorescence (405 nm) and Fluorescence (552 nm). **(A1-C1)** Imaging of yellow rectangular area in **(A-C)**. The yellow dotted line indicates the cells in the cambium being differentiated. **(D)** Differentiating cells in ray parenchyma. **(E)** Differentiating cells in vessel. **(F)** Differentiating cells in tracheid. **(D1-F1)** 3D fluorescence quantification diagram of yellow dot area in **(D-F)**. **(J)** Imaging of yellow rectangular area in **(H)**. **(J1-J3)** 3D fluorescence quantification diagram of yellow dot area in **(J)**. Scale bar:**(A-C)**50 μm, **(A1-C1)**10 μm, **(D-F)**5 μm. **(G-J)**25 μm.

It can be seen from the image of the flax stem sections (**Figure 14**) that the cambium still gradually differentiated to form cells after 60 days. After analyzing cells undergoing differentiation (**Figures 14A1–C1**), fluorescence could be observed in the cell corner and middle lamella of the whole cell (**Figures 14D–F**) during the cell formation process, indicating that lignin was generated and lignified simultaneously with cell differentiation. In the forming cells, cell corners were formed first and then the middle lamella. Higher fluorescence intensity was observed in cell corners (47a.u., 112a.u., 249a.u., 249a.u.) than that of the middle lamella (5a.u., 47a.u.) as shown in **Figure 14E1**.

**Figure 15:**
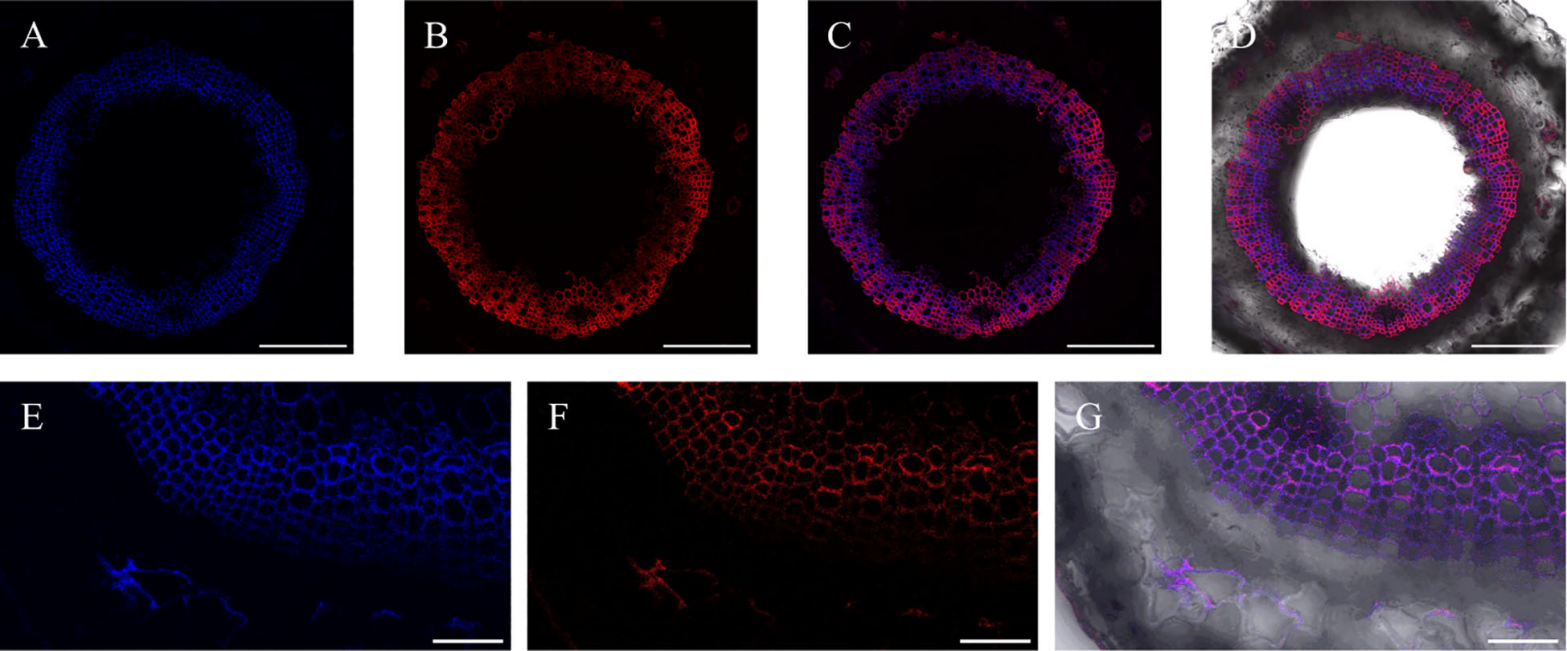
Incorporation of lignin precursors in bast fiber. **(A)** Lignin autofluorescence. **(B)** Fluorescence of labeled lignin. **(C)** Merger of A and B **(D)** Full view of autofluorescence and fluorescence of labeled lignin precursors in bright field. **(E)** Part view of lignin autofluorescence. **(F)** Part view of fluorescence of labeled lignin. **(G)** Merger of E and F. Scale bar, **(A-D)** 50 μm, **(E-G)** 10 μm.

At the same time, after incorporation of lignin precursors, fluorescence from alkynyl-labeled lignin can be clearly observed not only in the middle lamella and secondary cell walls, but also in the bast fiber (**Figure 15**), indicating that there was also a small amount of lignin formed in the bast fiber and cambium of flax.

## 4 Discussion

### 4.1 The structure of DHPs

**Figure 16:**
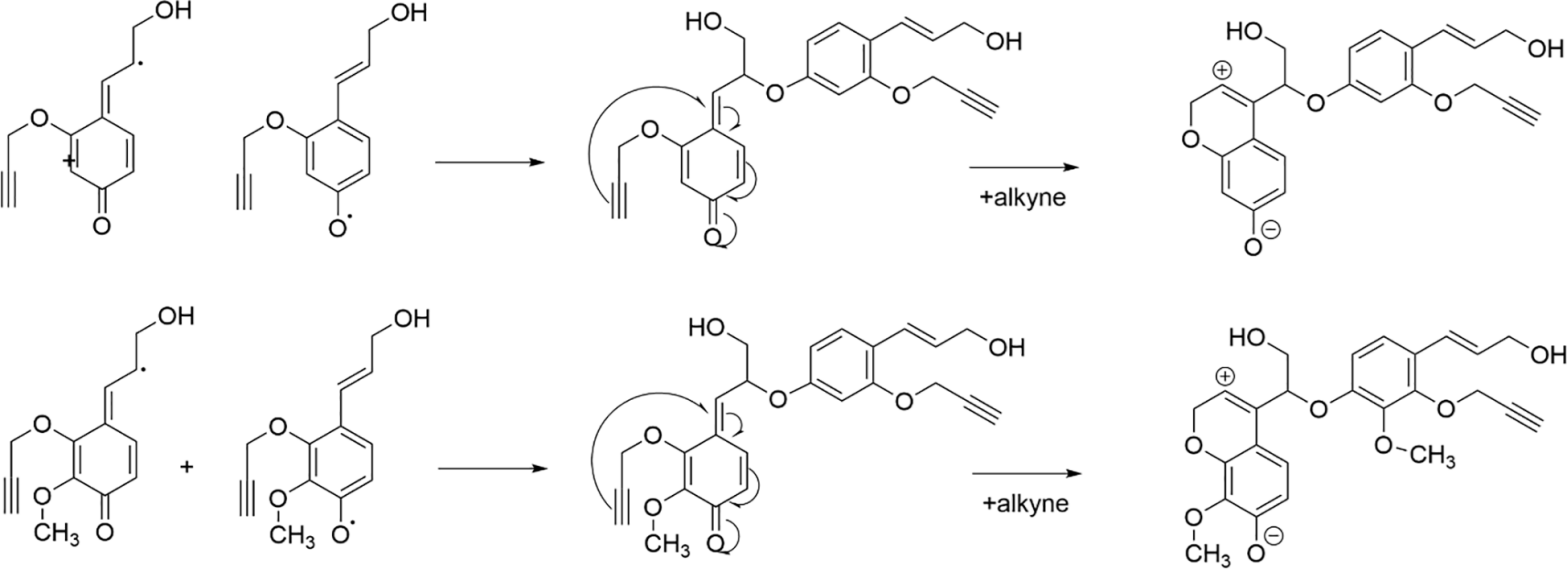
Pathway through which alkynes participate in the formation of novel *β*-ether bonds in DHPs.

All types of lignin DHPs contained propargyl group during the polymerization process and showed a structure similar to natural lignin, which indicated that lignin precursors labeled with propargyl groups were biocompatible. Similar to previous reports, H/GSH-DHP contained substructures of *β*-O-4, *β*-*5*, and *β*-*β* with the help of H_2_O_2_ and horseradish peroxidase (Harman-Ware et al., 2017; Yao et al., 2020). With the addition of lignin precursors (G and S), the content of *β*-O-4 increased, which was also found in another study (Harman-Ware et al., 2017). The absence of methoxy groups in the H lignin subunit resulted in the greatest unpaired electron density on the carbon nuclei, which was favorable for C-C formation (Yao et al., 2020). The results of DHPs also indicated that the content of *β*-O-4 in DHPs was relatively lower than those in natural lignin, while *β*-5 and *β-β* linkages were predominant, which was consistent with the previous studies (Landucci et al., 1998; Yao et al., 2020). When H_ALK_ was added, new signals were observed, from alkynyl and other new structures at 100.60/6.47 ppm (a), 107.93/6.37 ppm b), 95.14/6.57 ppm (c), 123.43/6.67 ppm (d), 82.80/5.65 ppm (e), 81.05/4.81 ppm (f), and 66.88/5.04 ppm (g) (**Figures 3**, **4**). One possible reason might be that part of the alkynyl group may react with quinone methide or take part in other side reactions (Pandey et al., 2015), resulting in a new type of linkage that has not been observed and identified in natural lignin (**Figure 16**). However, 15% H(H_ALK_)-DHP showed similar structures with H-DHP, indicating that precursors with low concentrations may not obviously change the structure of lignin. Furthermore, when H_ALK_ was used to synthesize DHP (100% H_ALK_-DHP), a signal from A_α_ (71.58/4.80 ppm) was not observed. However, when G, S, and H were all applied to synthesize DHP (GSH_ALK_-DHP), a signal from A_α_ could be detected. Due to the co-existence of G, S, and H precursors, the structural features of formed DHP were closer to natural lignin (Zeng et al., 2013). These results indicated that the alkynyl group was involved during DHP synthesis and precursors with alkynyl at low concentrations and these analogs showed the potential of lignification observation application *in vivo*.

### 4.2 The deposition of lignin in flax

Previous studies have shown that coniferyl alcohol analogs can undergo enzyme-induced oxidation and free radical coupling with naturally occurring lignin, thereby being incorporated into natural lignin polymers *in vivo* (Bukowski et al., 2014; Tobimatsu et al., 2014). In *Arabidopsis*, it is assumed that lignification begins at the attachment site of a lignin monomer, where lignin undergoes a series of reactions (Pandey et al., 2016). It was speculated that there is also such an attachment site in flax, where the units undergo free radical polymerization through the action of peroxidase and/or laccase to form lignin (Lion et al., 2017). When comparing the three histograms in **Figures 8A1, B1, C1**, coniferyl alcohol and *p*-coumary alcohol did not impact the fluorescence intensity after the click reaction. Under the same imaging conditions, the generation of fluorescence signals mainly depends on the click reaction and is a non-specific binding between azide dyes and alkynyl groups. From the image of the laser confocal microscope, it can be seen that the area labeled by Azide-fluor 545 was mainly in the middle lamella and cell wall of flax cells, which was the same as previous studies (Tobimatsu et al., 2014; Lion et al., 2017). From the fluorescence quantification of flax stem sections (**Figure 9**), the fluorescence intensity of flax stem sections increased with the increasing concentration of labeled lignin precursors, indicating that increased lignin precursor concentrations could promote deposition of the corresponding substance (**Figures 9A-F**).

With the increase in H_ALK_ concentration, the fluorescence intensity of labeled lignin gradually increased (**Figure 9**). However, the fluorescence intensity of G_ALK_ was first rapidly enhanced when the monomer concentration was increased from 1% to 50%. However, when the concentration further increased from 50% to 100%, the click fluorescence intensity did not significantly increase and even showed a certain downward trend, which may be caused by the difference in the deposition mode of the two different types of lignin in flax. In a previous study, azide tagged *p*-coumaryl alcohol and alkyne tagged coniferyl alcohol were synthesized and added to flax individually (Lion et al., 2017). The intensity of click fluorescence in the cells was the highest when the concentration of lignin precursors was the highest, which was similar to our results. Fluorescence intensity in plant slices cultivated with G-type lignin precursors tended to flatten as the concentration increased, while H-type lignin gradually increased. In our present study, the click fluorescence intensity in the stem section slices of flax cultivated with G_ALK_ or H_ALK_ was higher than that has been reported. It was speculated that the G_ALK_ and H_ALK_ synthesized in this study could be better incorporated by flax. At the same time, it was suggested that increasing the input of lignin precursors showed a relatively low impact on programmed cell death. Studies have shown that lignin deposition not only occurs after programmed cell death but also before (Smith et al., 2013). At the subcellular scale, the deposited lignin first appears in the cell corner and the middle lamella connecting adjacent plant cells, and then gradually on the cell wall (Wardrop, 1957; Saka and Thomas, 1982; Terashima et al., 1988; Donaldson, 1991, 1992, 2001; Boerjan et al., 2003; Smith et al., 2013).

As shown in **Figures 10C and D**, G_ALK_ or H_ALK_ were gradually incorporated over time, but their intensities were different. As the cultivation proceeded, the fluorescence intensity of H_ALK_ increased but was not as high as that of G_ALK_. The reason might be that flax is a type of gramineae, and the content of H-type lignin is lower than G-type lignin (Bonawitz and Chapple, 2010). Therefore, within the same cultivation time, the incorporation rate of G-type lignin was greater than that of H-type lignin. G_ALK_ reached its maximum fluorescence intensity at 24 hours, possibly due to saturation of the sections or limited click reagent. Due to the low content of H-lignin in the flax cells, the incorporation gradually increased with time, resulting in the fluorescence intensity reaching a maximum at 48 hours (**Figure 10D**). The synthesis of lignin is influenced by types of enzymes in plants, among which 4-coumarate:CoA ligase (4CL) limits the accumulation of lignin, and a decrease in 4CL content limits the availability of monolignol for lignin polymerization (Goudenhooft et al., 2019). During the process of lignin deposition, the depletion of C3H (*p*-coumarate-3-hydroxylase) activity leads to a decrease in H-lignin deposition (Anterola and Lewis, 2002). Due to the influence of various enzymes, the content of H-lignin is lower than that of G-lignin, resulting in different incorporations of the two types of lignin. Previous studies found that the deposition of G-type reached a maximum at 24 h and then started to decrease at 48 h as time went on. The fluorescence intensity of H-type reached a maximum at 12 h and then decreased (Lion et al., 2017), which was different from our results. The possible reason might be due to a different species and different chemical reporters applied.

The labeled flax cells showed stronger fluorescence in R and lower fluorescence in FT. In the root of *Arabidopsis*, autofluorescence in vascular bundles or xylem was higher than that in interfascicular fibers, but the click labeling was higher in interfascicular fibers (Pandey et al., 2016). The development of lignified cells includes cell expansion, biosynthesis of polysaccharides, lignification, and programmed cell death (depending on different cell types), which are closely coordinated in different regions (Lion et al., 2017; Obame et al., 2019). Earlier studies suggested that FT and R cells showed different lignification dynamics due to their various biological roles in developmental programs (Simon et al., 2018). Fiber tracheid exists in the xylem of plants and is used mainly for water transportation (Schuetz et al., 2014). Ray parenchyma (R) is widely distributed in plants, and its cells (**Figure 12**) show thin primary walls and more developed middle lamella, which can further develop into more specialized tissues (Simon et al., 2018). FTs mature and die rapidly, leaving cells with thick lignified walls, while R cells are alive in their mature and functional state and are free of thick lignified walls (Simon et al., 2018). Therefore, FT and R show different lignification trends (**Figure 12**). In plant cells, cell wall lignification occurs rapidly in one to two cells away from the cambium (Lion et al., 2017). The results showed that exogenous supply of monolignols and their analogs could be incorporated readily into lignified cell walls. A higher degree of incorporation could be observed in areas with lower autofluorescence, compared to areas already rich in lignin (**Figures 12D, E**), which was also found in the flax sections incorporated by the H_ALK_-type lignin (**Figures 12F, J**).

In the process of cell formation, the cell corner was formed first followed by the middle lamella and other regions (Baldacci-Cresp et al., 2020), which was also found in *Arabidopsis* (Lion et al., 2017). It is believed that during the process of cell differentiation and formation, lignin is formed, which could also be observed in **Figures 14D-F**. In similar studies, lignin biosynthesis starts with S1 formation (Lion et al., 2017), where lignin is synthesized. The newly formed lignin is first observed in cell corners and middle lamella, and then gradually diffuses into S1, S2, and S3 of secondary cell walls (Lion et al., 2017). Similar results were also found in H_ALK_ (**Figures 14G-J**). In *Arabidopsis*, when the concentration of lignin precursors was 20 μM, the degree of lignification in the secondary cell wall area was more intense than that in the cell corner and middle lamella (Pandey et al., 2016), which was in accordance with our research results. This result indicated that even though lignin was deposited in various regions of the cell, the deposition of lignin in the middle lamella was higher than that in the cell wall and other regions, which was in line with a previous report (Lion et al., 2017).

The bast fiber of flax is different from the xylem. The secondary cell wall of the bast fiber is rich in cellulose, while the content of lignin is relatively low (Day et al., 2005; del Río et al., 2011), and it also contains a higher content of H-type lignin than the xylem (Lion et al., 2017). Chemical analysis of the bast fiber and xylem cells of flax was performed previously (Day et al., 2005), and the results indicated that both the bast fiber and xylem of flax were rich in guaiacyl (G) units, and the S/G was lower than 0.5. Additionally, the lignin content in the bast fiber of flax was higher than that of the adjacent xylem. From **Figure 15**, it can be seen that relevant fluorescence of both natural lignin and artificially synthesized lignin can be observed in the phloem of flax (**Figures 15E-G**). Therefore, lignin deposits were observed not only in the xylem, but also in other parts such as the phloem in the plant. This is helpful for elucidating the lignin structure and valorization of plant bark (Neiva et al., 2020).

## 5 Conclusion

In the present study, alkyne groups were introduced at the ortho positions of lignin precursors, which were applied to investigate the lignification dynamics of flax. It was demonstrated that the lignin analog could be incorporated into the plant. Combined with CuAAC reaction, the lignification sites in the plant cell wall could be accurately located. The deposition pattern of lignin varies in different cells. In FT cells, the content of acetylene labeled lignin gradually decreased, while the content of natural lignin increased progressively. In R cells, the opposite trend was observed. During the process of cell formation, deposited lignin first appeared in the cell corner and the middle lamella, and then gradually appeared in the cell wall. When the cells matured, the highest content of lignin was found in the cell wall. These lignin analogs can be used to investigate the lignification of plants, which is beneficial for unraveling the mystery of how lignin deposits in plant cell walls.

## Data Availability

The original contributions presented in the study are included in the article/**Supplementary Material**. Further inquiries can be directed to the corresponding authors.

## References

[B1] Anterola A. M. Lewis N. G. (2002). Trends in lignin modification: a comprehensive analysis of the effects of genetic manipulations/mutations on lignification and vascular integrity. Phytochem. Rev. 61, 221–294. doi: 10.1016/S0031-9422(02)00211-X 12359514

[B2] Baldacci-Cresp F. Spriet C. Twyffels L. Blervacq A. S. Neutelings G. Baucher M.. (2020). A rapid and quantitative safranin-based fluorescent microscopy method to evaluate cell wall lignification. Plant J. 102, 1074–1089. doi: 10.1111/tpj.14675 31917878

[B3] Boerjan W. Ralph J. Baucher M. (2003). Lignin biosynthesis. Annu. Rev. Plant Biol. 54, 519–546. doi: 10.1146/annurev.arplant.54.031902.134938 14503002

[B4] Bonawitz N. D. Chapple C. (2010). The genetics of lignin biosynthesis: connecting genotype to phenotype. Annu. Rev. Genet. 44, 337–363. doi: 10.1146/annurev-genet-102209-163508 20809799

[B5] Bukowski N. Pandey J. L. Doyle L. Richard T. L. Anderson C. T. Zhu Y. (2014). Development of a clickable designer monolignol for interrogation of lignification in plant cell walls. Bioconjugate Chem. 25, 2189–2196. doi: 10.1021/bc500411u 25405515

[B6] Calvo-Flores F. G. Dobado J. A. (2010). Lignin as renewable raw material. ChemSusChem 3, 1227–1235. doi: 10.1002/cssc.201000157 20839280

[B7] Chiang V. L. (2006). Monolignol biosynthesis and genetic engineering of lignin in trees, a review. Environ. Chem. Lett. 4, 143–146. doi: 10.1007/s10311-006-0067-9

[B8] Day A. Ruel K. Neutelings G. Crônier D. David H. Hawkins S.. (2005). Lignification in the flax stem: evidence for an unusual lignin in bast fibers. Planta 222, 234–245. doi: 10.1007/s00425-005-1537-1 15968509

[B9] del Río J. C. Rencoret J. Gutiérrez A. Nieto L. Jiménez-Barbero J. Martínez Á.T. (2011). Structural characterization of guaiacyl-rich lignins in flax (Linum usitatissimum) fibers and shives. J. Agric. Food. Chem. 59, 11088–11099. doi: 10.1021/jf201222r 21905657

[B10] Donaldson L. A. (1991). Seasonal changes in lignin distribution during tracheid development in Pinus radiata D. Don. Wood Sci. Technol. 25, 15–24. doi: 10.1007/BF00195553

[B11] Donaldson L. A. (1992). Lignin distribution during latewood formation in pinus radiata D. Don. Iawa J. 13, 381–387. doi: 10.1163/22941932-90001291

[B12] Donaldson L. A. (2001). Lignification and lignin topochemistry — an ultrastructural view. Phytochem 57, 859–873. doi: 10.1016/S0031-9422(01)00049-8 11423137

[B13] Goudenhooft C. Bourmaud A. Baley C. (2019). Flax (Linum usitatissimum L.) fibers for composite reinforcement: exploring the link between plant growth, cell walls development, and fiber properties. Front. Plant Sci. 10. doi: 10.3389/fpls.2019.00411 PMC6456768 31001310

[B14] Guo Y. Zhou J. Wen J. Sun G. Sun Y. (2015). Structural transformations of triploid of Populus tomentosa Carr. lignin during auto-catalyzed ethanol organosolv pretreatment. Ind. Crops Prod. 76, 522–529. doi: 10.1016/j.indcrop.2015.06.020

[B15] Halina P. K. Arora A. Gupta A. Saeed M. A. Niedzwiecki L. Andrews G.. (2020). Biocoal - Quality control and assurance. Biomass Bioenergy 135, 105509. doi: 10.1016/j.biombioe.2020.105509

[B16] Harman-Ware A. E. Happs R. M. Davison B. H. Davis M. F. (2017). The effect of coumaryl alcohol incorporation on the structure and composition of lignin dehydrogenation polymers. Biotechnol. Biofuels 10, 281–292. doi: 10.1186/s13068-017-0962-2 29213321 PMC5707875

[B17] Karmanov A. P. Derkacheva O. Y. (2013). Application of fourier transform infrared spectroscopy for the study of lignins of herbaceous plants. Russ. J. Bioorg Chem. 39, 677–685. doi: 10.1134/S1068162013070066

[B18] Kim H. Ralph J. (2010). Solution-state 2D NMR of ball-milled plant cell wall gels in DMSO-d6/pyridine-d5. Org. Biomol. Chem. 8, 576–591. doi: 10.1039/B916070A 20090974 PMC4070321

[B19] Landucci L. L. Ralph S. A. Hammel K. E. (1998). ^13^C NMR characterization of guaiacyl, guaiacyl/syringyl and syringyl dehydrogenation polymers. Holzforschung 52, 160–170. doi: 10.1515/hfsg.1998.52.2.160

[B20] Lavis L. D. Chao T. Y. Raines R. T. (2006). Fluorogenic label for biomolecular imaging. ACS Chem. Biol. 1, 252–260. doi: 10.1021/cb600132m 17163679 PMC2862228

[B21] Lim R. K. V. Lin Q. (2010). Bioorthogonal chemistry: a covalent strategy for the study of biological systems. Sci. China Chem. 53, 61–70. doi: 10.1007/s11426-010-0020-4 20694042 PMC2914326

[B22] Lion C. Simon C. Huss B. Blervacq A. S. Tirot L. Toybou D.. (2017). BLISS: A bioorthogonal dual-labeling strategy to unravel lignification dynamics in plants. Cell Chem. Biol. 24, 326–338. doi: 10.1080/15592324.2017.1359366 28262560

[B23] Liu C. J. (2012). Deciphering the enigma of lignification: precursor transport, oxidation, and the topochemistry of lignin assembly. Mol. Plant 5, 304–317. doi: 10.1093/mp/ssr121 22307199

[B24] Liu Y. Hu T. Wu Z. Zeng G. Huang D. Shen Y.. (2014). Study on biodegradation process of lignin by FTIR and DSC. Environ. Sci. pollut. Res. 21, 14004–14013. doi: 10.1007/s11356-014-3342-5 25037100

[B25] Liu Q. Luo L. Zheng L. (2018). Lignins: biosynthesis and biological functions in plants. Int. J. Mol. Sci. 19, 335. doi: 10.3390/ijms19020335 29364145 PMC5855557

[B26] Lou H. Lai H. Wang M. Pang Y. Yang D. Qiu X.. (2013). Preparation of lignin-based superplasticizer by graft sulfonation and investigation of the dispersive performance and mechanism in a cementitious system. Ind. Eng. Chem. Res. 52, 16101–16109. doi: 10.1021/ie402169g

[B27] Luterbacher J. S. Martin Alonso D. Dumesic J. A. (2014). Targeted chemical upgrading of lignocellulosic biomass to platform molecules. Green Chem. 16, 4816–4838. doi: 10.1039/C4GC01160K

[B28] McIntosh A. L. Atshaves B. P. Huang H. Gallegos A. M. Kier A. B. Schroeder F. (2008). Fluorescence techniques using dehydroergosterol to study cholesterol trafficking. Lipids 43, 1185–1208. doi: 10.1007/s11745-008-3194-1 18536950 PMC2606672

[B29] Neiva D. M. Rencoret J. Marques G. Gutiérrez A. Gominho J. Pereira H.. (2020). Lignin from tree barks: chemical structure and valorization. ChemSusChem 13, 4537–4547. doi: 10.1002/cssc.202000431 32395900 PMC7540371

[B30] Obame S. N. Ziegler-Devin I. Safou-Tchima R. Brosse N. (2019). Homolytic and heterolytic cleavage of β-ether linkages in hardwood lignin by steam explosion. J. Agric. Food. Chem. 67, 5989–5996. doi: 10.1021/acs.jafc.9b01744 31062970

[B31] Pandey J. L. Kiemle S. N. Richard T. L. Zhu Y. Cosgrove D. J. Anderson C. T. (2016). Investigating biochemical and developmental dependencies of lignification with a click-compatible monolignol analog in *arabidopsis thaliana* stems. Front. Plant Sci. 7. doi: 10.3389/fpls.2016.01309 PMC5005335 27630649

[B32] Pandey J. L. Wang B. Diehl B. G. Richard T. L. Chen G. Anderson C. T. (2015). A versatile click-compatible monolignol probe to study lignin deposition in plant cell walls. PloS One 10, e0121334. doi: 10.1371/journal.pone.0121334 25884205 PMC4401456

[B33] Ragauskas A. J. Williams C. K. Davison B. H. Britovsek G. Cairney J. Eckert C. A.. (2006). The path forward for biofuels and biomaterials. Science 311, 484–489. doi: 10.1126/science.1114736 16439654

[B34] Ralph J. Brunow G. Harris P. J. Dixon R. A. Schatz P. F. Boerjan W. (2008). Lignification: are Lignins Biosynthesized via simple Combinatorial Chemistry or via Proteinaceous Control and Template Replication? Recent Adv. Polyphenol. Res. 1, 36–66. doi: 10.1002/9781444302400

[B35] Ralph J. Lundquist K. Brunow G. Lu F. Kim H. Schatz P. F.. (2004). Lignins: Natural polymers from oxidative coupling of 4-hydroxyphenylpropanoids. Phytochem. Rev. 3, 29–60. doi: 10.1023/B:PHYT.0000047809.65444

[B36] Sadeghifar H. Ragauskas A. (2020). Lignin as a UV light blocker-a review. Polymers(Basel) 12, 1134. doi: 10.3390/polym12051134 32429134 PMC7284897

[B37] Saka S. Thomas R. J. (1982). A study of lignification in loblolly pine tracheids by the SEM-EDXA technique*. Wood Sci. Technol. 16, 167–179. doi: 10.1007/BF00353866

[B38] Schuetz M. Benske A. Smith R. A. Watanabe Y. Tobimatsu Y. Ralph J.. (2014). Laccases direct lignification in the discrete secondary cell wall domains of protoxylem. Plant Physiol. 166, 798–807. doi: 10.1104/pp.114.245597 25157028 PMC4213109

[B39] Simon C. Lion C. Huss B. Blervacq A. S. Spriet C. Guérardel Y.. (2017). BLISS: Shining a light on lignification in plants. Plant Signaling Behav. 12, e1359366. doi: 10.1080/15592324.2017.1359366 PMC5616161 28786751

[B40] Simon C. Spriet C. Hawkins S. Lion C. (2018). Visualizing lignification dynamics in plants with click chemistry: dual labeling is BLISS! J. Visual. Experiments 131, e56947. doi: 10.3791/56947 PMC5908702 29443107

[B41] Sletten E. M. Bertozzi C. R. (2009). Bioorthogonal chemistry: fishing for selectivity in a sea of functionality. Angew. Chem. Int. Ed. 48, 6974–6998. doi: 10.1002/anie.200900942 PMC2864149 19714693

[B42] Smith R. A. Schuetz M. Roach M. Mansfield S. D. Ellis B. Samuels L. (2013). Neighboring parenchyma cells contribute to *arabidopsis* xylem lignification, while lignification of interfascicular fibers is cell autonomous. Plant Cell. 25, 3988–3999. doi: 10.1105/tpc.113.117176 24096341 PMC3877792

[B43] Sun Z. Fridrich B. de Santi A. Elangovan S. Barta K. (2018). Bright side of lignin depolymerization: toward new platform chemicals. Chem. Rev. 118, 614–678. doi: 10.1021/acs.chemrev.7b00588 29337543 PMC5785760

[B44] Terashima N. Fukushima K. Sano Y. Takabe K. (1988). Heterogeneity in formation of lignin X. Visualization of lignification process in differentiating xylem of pine by microautoradiography*). Holzforschung 42, 347–350. doi: 10.1515/hfsg.1988.42.6.347

[B45] Tobimatsu Y. de Wouwer D. V. Allen E. Kumpf R. Vanholme B. Boerjan W.. (2014). A click chemistry strategy for visualization of plant cell wall lignification. Chem. Commun. 50, 12262–12265. doi: 10.1039/C4CC04692G 25180250

[B46] Umezawa T. (2009). The cinnamate/monolignol pathway. Phytochem. Rev. 9, 1–17. doi: 10.1007/s11101-009-9155-3

[B47] Vanholme R. Cesarino I. Rataj K. Xiao Y. G. Sundin L. Goeminne G.. (2013). Caffeoyl shikimate esterase (CSE) is an enzyme in the lignin biosynthetic pathway in *arabidopsis* . Science 341, 1103–1106. doi: 10.1126/science.1241602 23950498

[B48] Vanholme R. Demedts B. Morreel K. Ralph J. Boerjan W. (2010). Lignin biosynthesis and structure. Plant Physiol. 153, 895–905. doi: 10.1104/pp.110.155119 20472751 PMC2899938

[B49] Wang Z. Qiu S. Hirth K. Cheng J. Wen J. Li N.. (2019). Preserving both lignin and cellulose chemical structures: flow-through acid hydrotropic fractionation at atmospheric pressure for complete wood valorization. ACS Sustain. Chem. Eng. 7, 10808–10820. doi: 10.1021/acssuschemeng.9b01634

[B50] Wardrop A. B. (1957). The phase of lignification in the differentiation of wood fibers. Tappi 40, 225–243.

[B51] Watson P. Jones A. T. Stephens D. J. (2005). Intracellular trafficking pathways and drug delivery: fluorescence imaging of living and fixed cells. Adv. Drug Deliv. Rev. 57, 43–61. doi: 10.1016/j.addr.2004.05.003 15518920

[B52] Yao L. Yang H. Yoo C. G. Chen C. Meng X. Jun D.. (2020). A mechanistic study of cellulase adsorption onto lignin. Green Chem. 23, 1–7. doi: 10.1039/D0GC02463E

[B53] Zeng J. Helms G. L. Gao X. Chen S. (2013). Quantification of wheat straw lignin structure by comprehensive NMR analysis. J. Agric. Food. Chem. 61, 10848–10857. doi: 10.1021/jf4030486 24143908

[B54] Zhang Y. Naebe M. (2021). Lignin: a review on structure, properties, and applications as a light-colored UV absorber. ACS Sustain. Chem. Eng. 9, 1427–1442. doi: 10.1021/acssuschemeng.0c06998

