# Supplementary material for: Visualization of lignification in flax stem cell walls with novel click-compatible monolignol analogs
Source: Front Plant Sci. 2024 Aug 19;15:1423072. doi: 10.3389/fpls.2024.1423072 (PMC11366659; doi:10.3389/fpls.2024.1423072)
Supplement: Supplementary file 1 [file DataSheet1.docx]

Supplementary Material

# Supplementary Figures


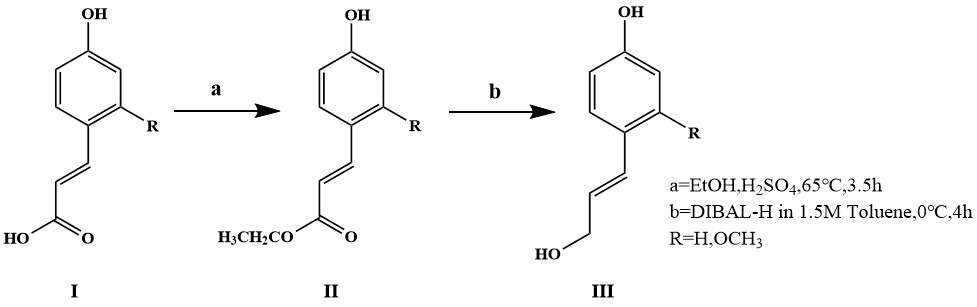


**Supplementary Figure S1.** Synthetic method of *p*CA and CA and SA.


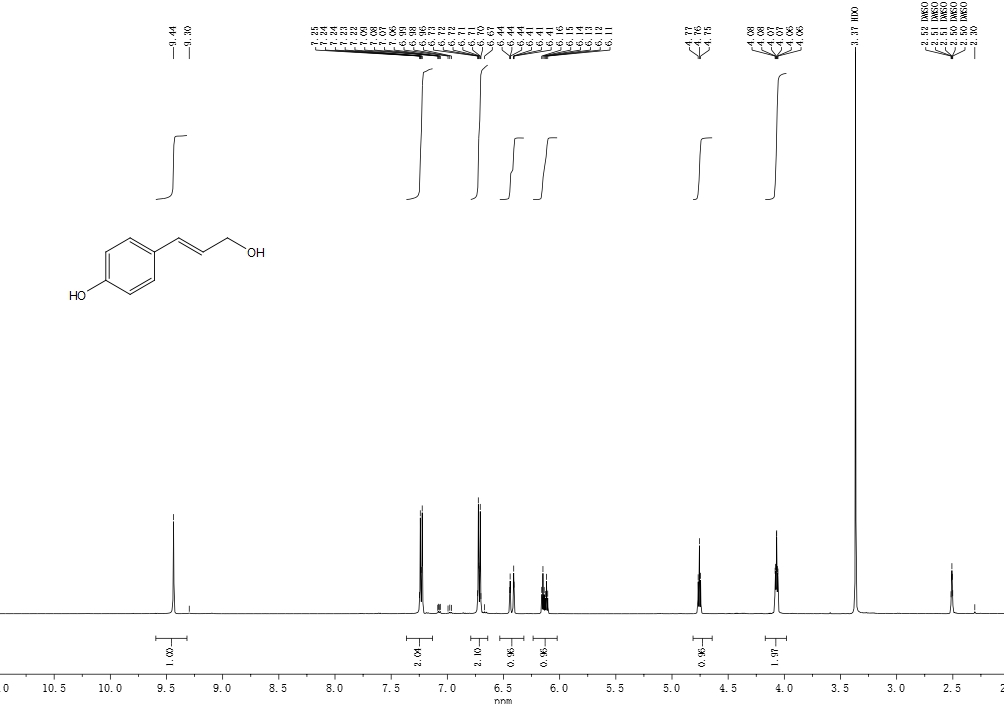


**Supplementary Figure S2.** ^1^H-NMR spectrum of *p*-Coumaric Acid Ethyl Ester (Ⅲ).

P-Hydroxybenzoic acid (5.8g) was put into a three necked flask with 25ml of ethanol and 15ml of n-hexane. About 0.5ml of concentrated sulfuric acid was added through a constant pressure funnel. After heating and refluxing for 3h, 10% sodium carbonate was added to adjust the pH to neutral. Aftering adding 30ml of ethylacetate, and then washed by saturated sodium chloride twice until the color of the water layer was clear. The collected organic layer was dried with anhydrous sodium sulfate for 12 hours, which then was evaporated under reduced pressure, and the residue was separated by column chromatography. The mobile phase was n-hexane/ethylacetate (2:1, v/v). Finally, the target component was evaporated under reduced pressure. After recrystallization, a white crystal with a yield of 89% and a melting point of 64.5~67.8 ℃ was obtain, which was ethyl coumaric acid. ^1^H NMR (500 MHz, DMSO-d_6_) δ 9.44 (s, 1H), 7.32 – 7.17 (m, 2H), 6.79 – 6.62 (m, 2H), 6.43 (dt, J = 15.8, 1.6 Hz, 1H), 6.13 (dt, J = 15.9, 5.4 Hz, 1H), 4.76 (t, J = 5.5 Hz, 1H), 4.07 (td, J = 5.5, 1.6 Hz, 2H).


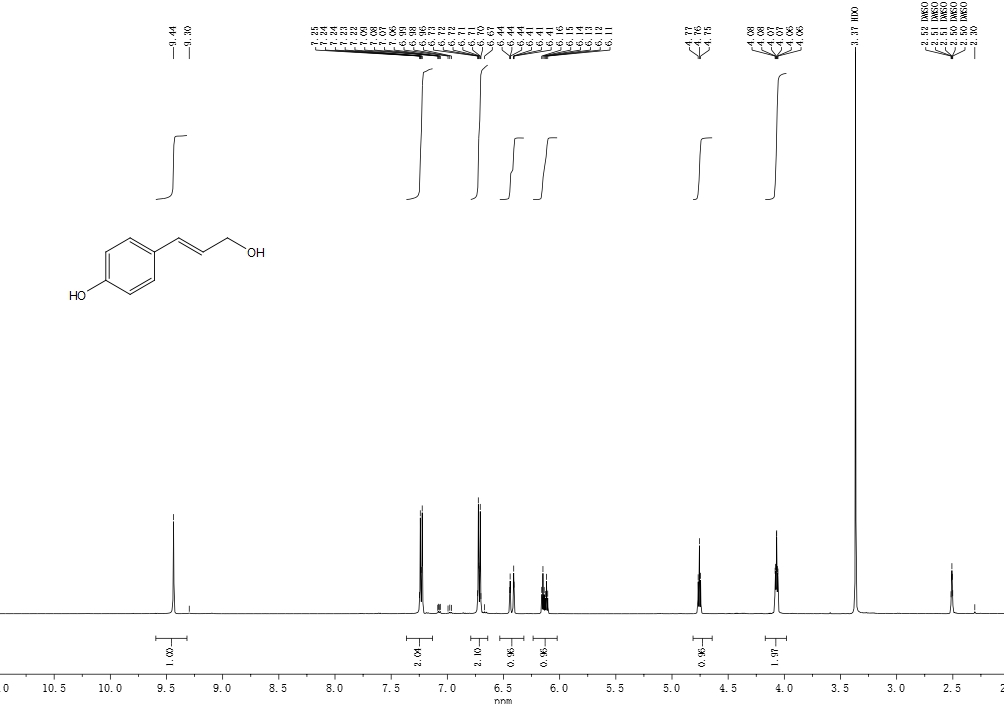


**Supplementary Figure S3.** ^1^H-NMR spectrum of *p*CA(Ⅲ).

Product Ⅱ (1.44g) was added into a three necked flask with 20ml of tetrahydrofuran for dissolution under nitrogen. Around 20ml of DIBAL-H (Diisobutylaluminum hydride) 1.5M in toluene was joined at 0 ℃ into a low temperature reaction bath through a constant pressure funnel within 30min. After 4 hours, 2ml of methanol was added to terminate the reaction. After mixing with 30mL saturated Potassium sodium tartrate solution and 40ml ethyl acetate, and stirring at room temperature for 1h, the ester layer was separated. The water layer was extracted with 20ml ethyl acetate. The collected ester layer was dried with anhydrous sodium sulfate overnight. After filtration, vacuum evaporation, and recrystallize with n-hexane/ether (2:1, V/V), *p*CA was obtained with 80% yield, and the melting point was 83.3~85.5 ℃. ^1^H NMR (500 MHz, DMSO-d_6_) δ 9.44 (s, 1H), 7.32 – 7.17 (m, 2H), 6.79 – 6.62 (m, 2H), 6.43 (dt, J = 15.8, 1.6 Hz, 1H), 6.13 (dt, J = 15.9, 5.4 Hz, 1H), 4.76 (t, J = 5.5 Hz, 1H), 4.07 (td, J = 5.5, 1.6 Hz, 2H).


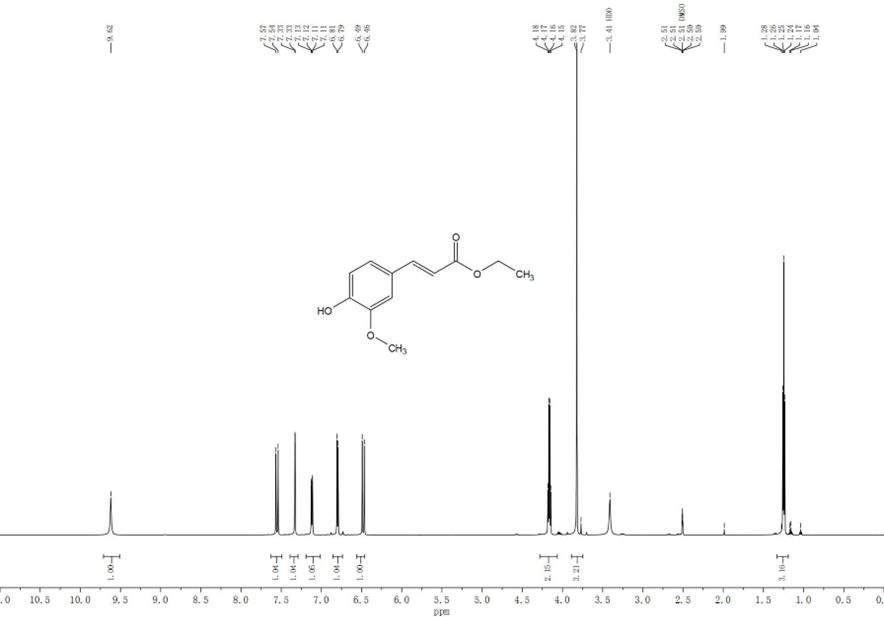


**Supplementary Figure S4.** ^1^H-NMR spectrum of Ferulic Acid Ethyl Ester (Ⅱ).

Synthesis of Ferulic Acid Ethyl Ester was the same as that of *p*-Coumaric Acid Ethyl Ester. Ethyl ferulate, ^1^H NMR (600 MHz, DMSO-d6) δ 9.62 (s, 1H), 7.55 (d, J = 15.9 Hz, 1H), 7.33 (d, J = 2.0 Hz, 1H), 7.12 (dd, J = 8.1, 1.9 Hz, 1H), 6.80 (d, J = 8.1 Hz, 1H), 6.48 (d, J = 15.9 Hz, 1H), 4.16 (q, J = 7.1 Hz, 2H), 3.82 (s, 3H), 1.25 (t, J = 7.1 Hz, 3H).


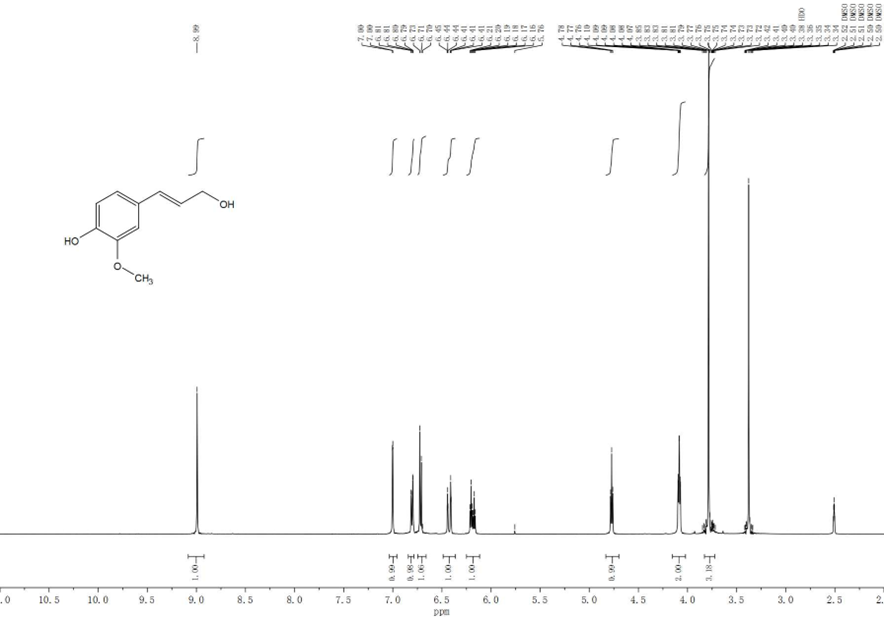


**Supplementary Figure S5.** ^1^H-NMR spectrum of coniferyl alcohol (Ⅲ).

Synthesis of CA was the same as that of *p*CA. ^1^H NMR (600 MHz, DMSO-d_6_) δ 9.01 (s, 1H), 7.01 (d, J = 2.0 Hz, 1H), 6.81 (dd, J = 8.1, 2.0 Hz, 1H), 6.72 (d, J = 8.1 Hz, 1H), 6.43 (dt, J = 15.8, 1.8 Hz, 1H), 6.19 (dt, J = 15.9, 5.4 Hz, 1H),4.75(s,1H）,4.09 (dd, J = 5.5, 1.6 Hz, 2H), 3.79 (s, 3H).


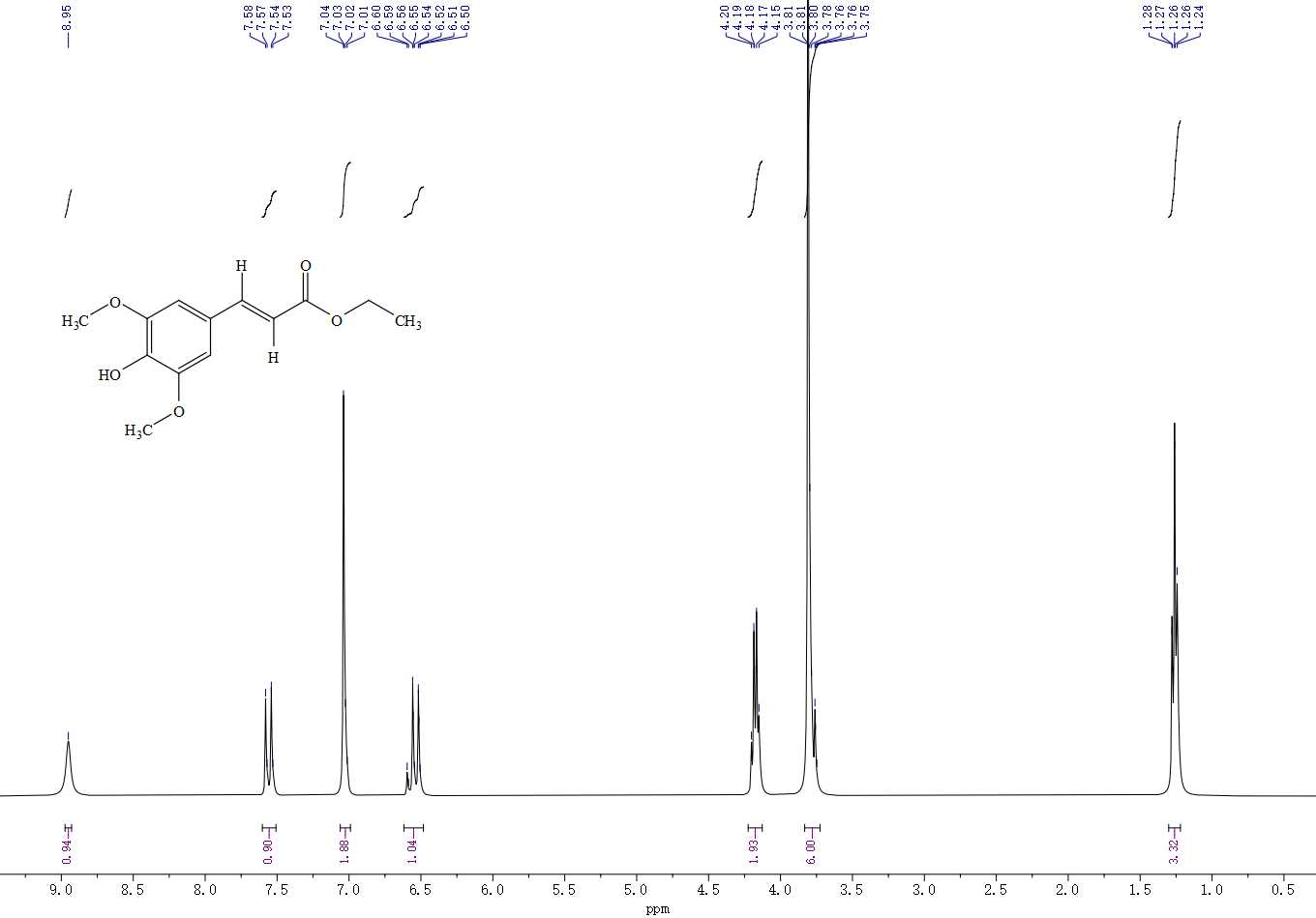


**Supplementary Figure S6.** ^1^H-NMR spectrum of Sinapic acid Ethyl Ester (Ⅱ).

The synthesis procedure of Sinapic acid Ethyl Ester is the same as that of p-Coumaric Acid Ethyl Ester. ^1^H NMR (400 MHz, DMSO-d_6_) δ 8.95 (s, 1H), 7.55 (dd, J = 15.9, 4.1 Hz, 1H), 7.04 (d, J = 1.6 Hz, 2H), 6.62 – 6.48 (m, 1H), 4.17 (dd, J = 10.0, 4.2 Hz, 2H), 3.83 – 3.73 (m, 6H), 1.30 – 1.22 (m, 3H).


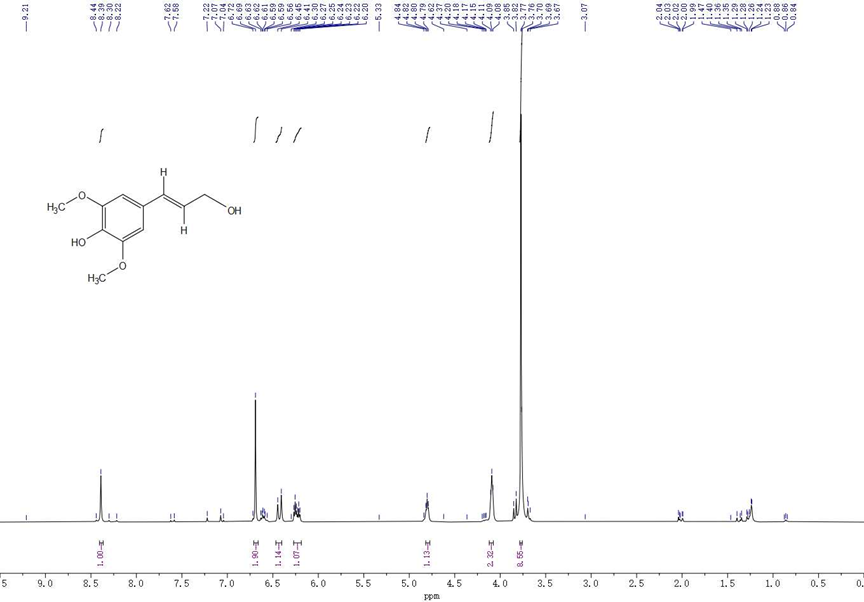


**Supplementary Figure S7.** ^1^H-NMR spectrum of Sinapyl alcohol (Ⅲ).

Sinapic acid Ethyl Ester (0.3g) was mixed with 14.75mL toluene under N_2_. Then, diisobutyl aluminium hydride (DIBAL-H) 1.5M in toluene was added at 0 ℃, and stirred for 1h. Anhydrous ethanol was added to quench the reaction until no bubbles were generated. After vacuum evaporation at 40 ℃, EA and saturated Potassium sodium tartrate were joined to extract and separate the solution. The ester layer was collected and dried over anhydrous Na_2_SO_4_. After filtration and vacuum evaporation, the solution was separated by column chromatography (n-hexane/EA, 2:1, v/v). Finally, after recrystallization with methylene chloride/petroleum ether, the target product was obtained with a yield of 83.98% and a melting point of 65.6~68.3 ℃. ^1^H NMR (400 MHz, DMSO-d_6_) δ 8.39 (s, 1H), 6.69 (s, 2H), 6.43 (d, J = 15.8 Hz, 1H), 6.24 (dt, J = 15.8, 5.3 Hz, 1H), 4.80 (t, J = 5.4 Hz, 1H), 4.09 (t, J = 5.2 Hz, 2H), 3.77 (d, J = 3.4 Hz, 9H).


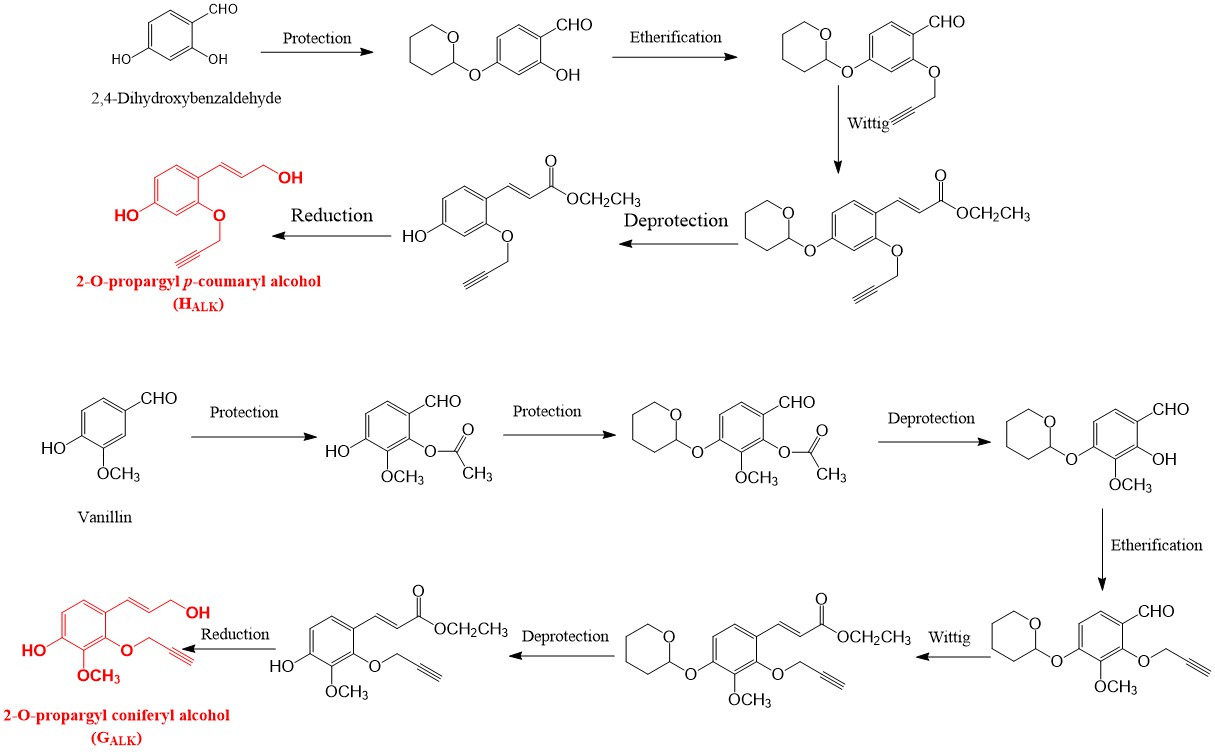


**Supplementary Figure S8.** Synthetic method of 2-O-PPA (H_ALK_).


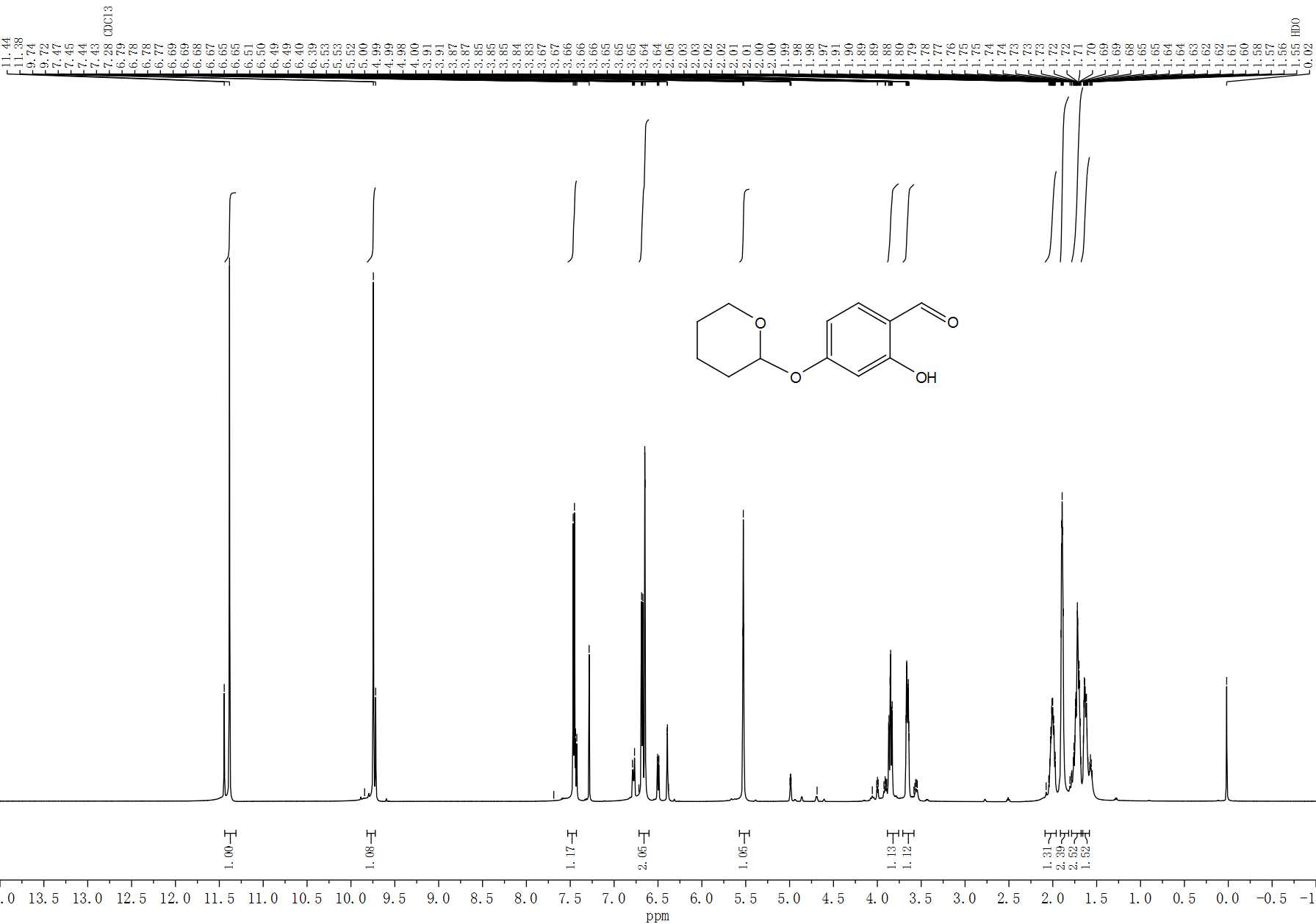


**Supplementary Figure S9.** ^1^H-NMR spectrum of 4-O-tetrahydropyranyl-2-hydroxy benzaldehyde (II).

2,4-dihydroxybenzaldehyde (0.967g) and of 2,6-Dimethylpyridinium *p*-Toluenesulfonate (0.088g) were dissolved in a three necked flask with 25mL of dichloromethane at room temperature. 3,4-dihydro-2H-pyran (1.3ml) was added dropwise by a constant pressure funnel, and stirred for 1.5 hours. Then, 70 ml of ether and 50ml of distilled water were added. The ether layer was collected and washed with 50ml of distilled water, which then was dried with anhydrous sodium sulfate for 12 hours. After filtration, vacuum evaporation, the target product was separated through a chromatographic column, with a mobile phase of n-hexane/ethyl acetate (2:1, v/v). The target product was vacuum evaporated, then white crystalline product II was precipitated by crystallization with ether and n-hexane, with a yield of 60% and a melting point of 57.2~59.6 ℃. ^1^H NMR (600 MHz, Chloroform-d) δ 11.38 (s, 1H), 9.73 (d, J = 15.8 Hz, 1H), 7.45 (dd, J = 16.1, 8.5 Hz, 1H) , 6.68 (dd, J = 8.7, 2.3 Hz, 1H), 6.65 (d, J = 2.2 Hz, 1H), , 5.53 (t, J = 3.2 Hz, 1H), 3.85 (td, J = 10.7, 3.1 Hz, 1H), 3.66 (ddt, J = 11.5, 4.3, 2.4 Hz, 1H), 2.00 (dddt, J = 14.1, 10.3, 7.7, 3.9 Hz, 1H), 1.89 (dt, J = 7.9, 3.8 Hz, 2H), 1.80 – 1.70 (m, 1H), 1.73 – 1.66 (m, 1H), 1.63 (dp, J = 12.9, 4.3, 3.9 Hz, 1H).


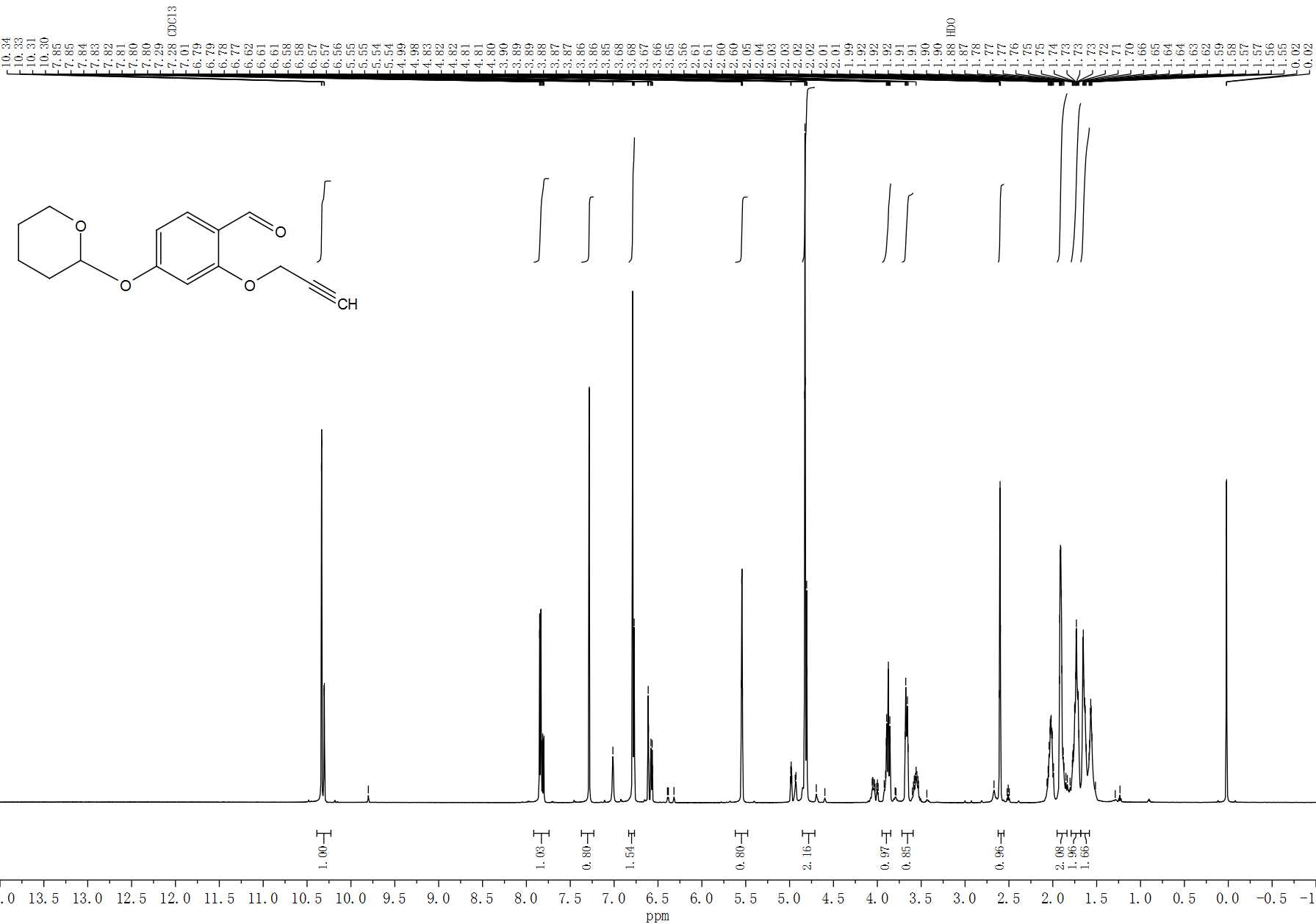


**Supplementary Figure S10.** ^1^H-NMR spectrum of 4-O-tetrahydropyranyl-2-propargyl benzaldehyde (Ⅲ).

Product II (2.22g) was dissolved in a bottle containing 5ml of N, N-dimethylformamide. Then, 1.506g of potassium carbonate was joined and stirred at room temperature for 20min. The solution was changed from colorless to light yellow. Propargyl bromide (1.188g) was added by constant pressure funnel. After stirring for 4 hours in 40 ℃ oil bath, 30ml of ethyl acetate was added for extraction, and then distilled water (3 × 30ml) was added. The ester layer was collected and dried overnight with anhydrous sodium sulfate. After filtration, vacuum evaporation, yellow white solid III was obtained, with a yield of 80% and a melting point of 43.4~45.8 ℃. ^1^H NMR (600 MHz, Chloroform-d) δ 10.32 (dd, J = 18.2, 1.8 Hz, 1H), 7.83 (ddd, J = 19.6, 8.4, 1.8 Hz, 1H), 6.81 – 6.76 (m, 1H), 6.63 – 6.55 (m, 1H), 5.54 (q, J = 2.7 Hz, 1H), , 4.82 (dt, J = 11.5, 2.2 Hz, 2H), 3.87 (td, J = 10.7, 2.4 Hz, 1H), 3.67 (dd, J = 10.3, 5.1 Hz, 1H) , 2.60 (q, J = 2.5 Hz, 1H), 2.07 – 1.97 (m, 1H), 1.94 – 1.80 (m, 1H), 1.79 – 1.72 (m, 1H), 1.72 (dd, J = 9.2, 3.6 Hz, 1H), 1.67 – 1.61 (m, 1H), 1.61 – 1.54 (m, 1H).


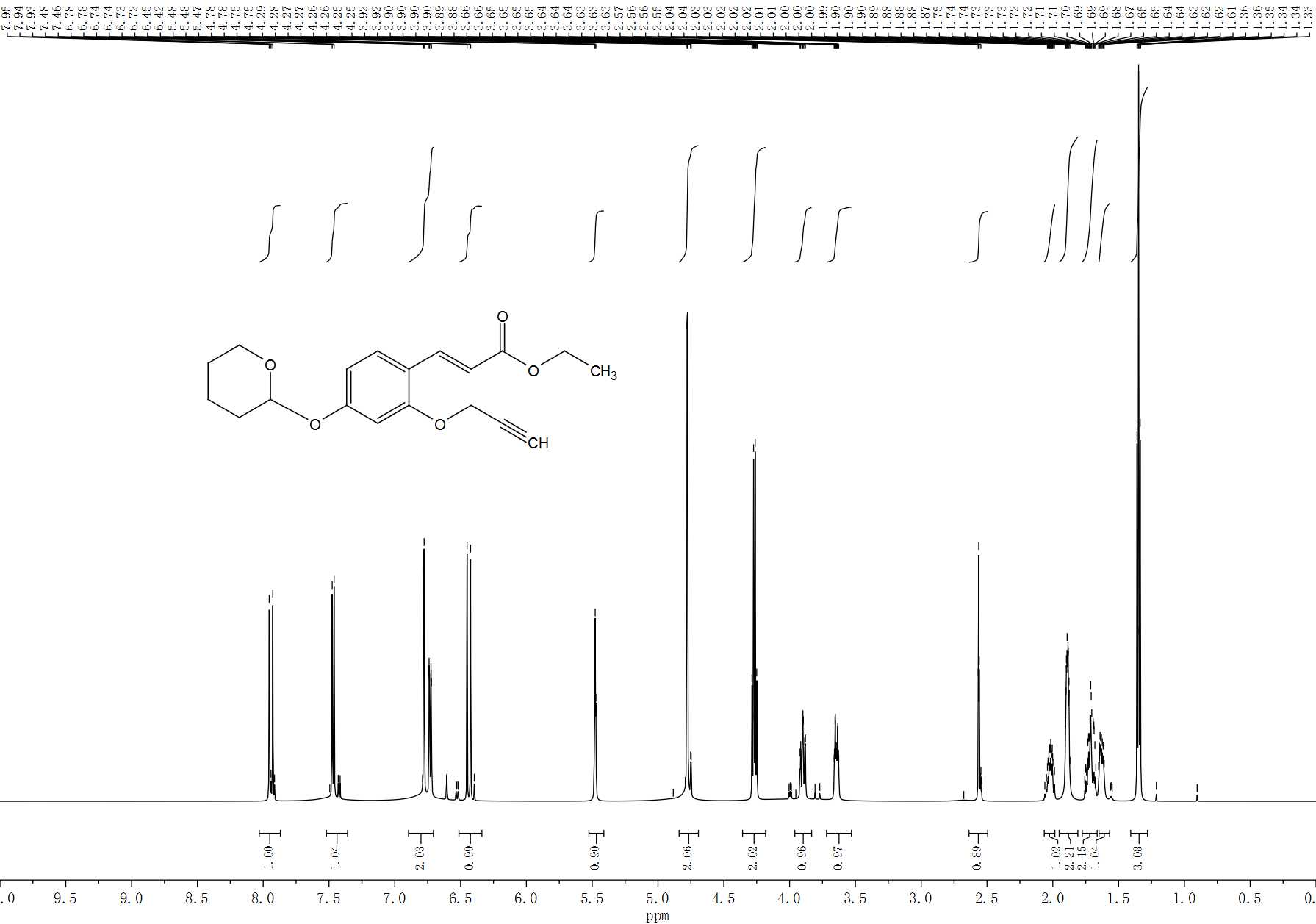


**Supplementary Figure S11.** ^1^H-NMR spectrum of 4-O-tetrahydropyranyl-2-propargyl cinnamate ethyl ester (IV).

Triethyl phosphonoacetate (5.7ml) was added to a three necked flask with 40ml of tetrahydrofuran under nitrogen. Then, 1.1g of NaH was joined in a low temperature bath at 0 ℃. The solution was stirred for 15min. Reactant III was dissolved in 10ml of tetrahydrofuran and dropped dropwise through a constant pressure funnel, followed by a reaction at room temperature for 12 hours. Afterwards, 25ml of distilled water was added. After the water layer was separated, saturated sodium bicarbonate was added to collect the organic layer. Then tert-butyl methyl ether (3 × 30ml) was added to extract the water layer. The collected organic layer was dried overnight with anhydrous sodium sulfate, which then was filtered and vacuum evaporated. The target product was separated by chromatography with mobile phase of n-hexane/Methyl tert-butyl ether (10:1 to 5:1, v/v). The target product was evaporated under reduced pressure, and white crystal product Ⅳ was obtained with yield of 78%, melting point of 58.3~60.7 ℃. ^1^H NMR (600 MHz, Chloroform-d) δ 7.945 (d, J = 16.1 Hz, 1H), 7.49 – 7.40 (m, 1H), 6.78 (d, J = 2.3 Hz, 1H), 6.73 (dd, J = 8.6, 2.3 Hz, 1H), 6.44 (d, J = 16.1 Hz, 1H), 5.48 (t, J = 3.2 Hz, 1H), 4.78 (d, J = 2.4 Hz, 2H), 4.27 (q, J = 7.1 Hz, 2H), 3.95 (s, 1H), 3.90 (ddd, J = 11.4, 9.9, 3.1 Hz, 1H), 3.65 (ddt, J = 10.3, 4.4, 2.2 Hz, 1H), ,2.58 – 2.53 (m, 1H), 2.08 – 1.97 (m, 1H), 1.89 (s, 1H), 1.94 – 1.85 (m, 1H), 1.77 – 1.58 (m, 3H), 1.35 (t, J = 7.1 Hz, 3H).


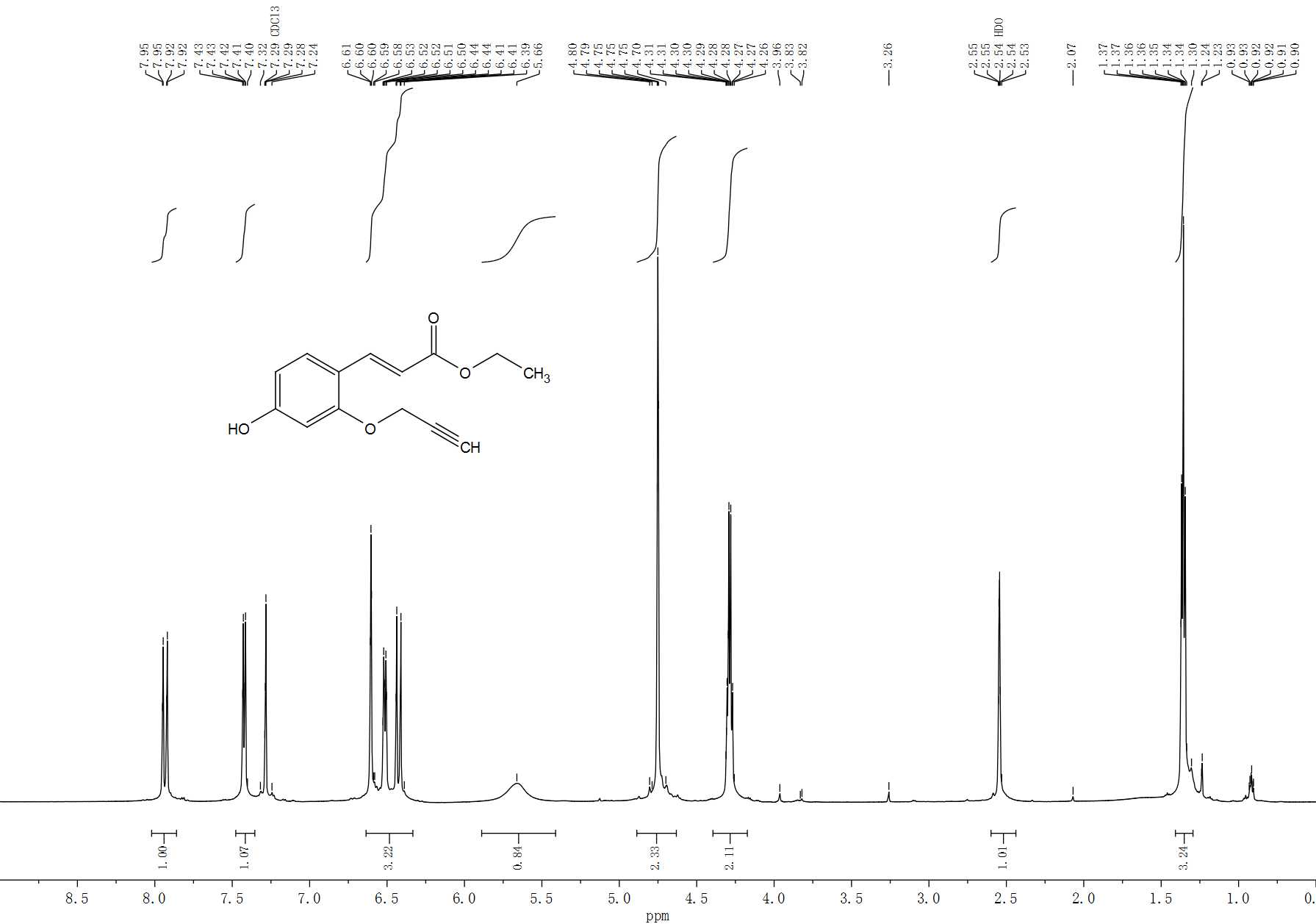


**Supplementary Figure S12.** ^1^H-NMR spectrum of ethyl 4-hydroxy-2-propargyl cinnamate ethyl ester (V).

Triethyl phosphonoacetate (5.7ml) was added to a three necked flask with 40ml of tetrahydrofuran under nitrogen, after which 1.1g of NaH was joined in a low temperature bath at 0 ℃. The solution was stirred for 15min. Product III was dissolved in 10ml of tetrahydrofuran and dropped dropwise through a constant pressure funnel, followed by a reaction at room temperature for 12 hours. Then, 25ml of distilled water was added. After the water layer was separated, 25ml of saturated sodium bicarbonate was added to collect the organic layer. Then tert-butyl methyl ether (3 × 30ml) was added to extract the water layer. The collected organic layer was dried overnight with anhydrous sodium sulfate, which then was filtered and vacuum evaporated. The target product was separated by chromatography with mobile phase of n-hexane/Methyl tert-butyl ether (10:1 to 5:1, v/v). The target product was evaporated under reduced pressure, and white crystal product Ⅳ was obtained with yield of 78%, melting point of 58.3~60.7 ℃. ^1^H NMR (600 MHz, Chloroform-d) δ 7.945 (d, J = 16.1 Hz, 1H), 7.49 – 7.40 (m, 1H), 6.78 (d, J = 2.3 Hz, 1H), 6.73 (dd, J = 8.6, 2.3 Hz, 1H), 6.44 (d, J = 16.1 Hz, 1H), 5.48 (t, J = 3.2 Hz, 1H), 4.78 (d, J = 2.4 Hz, 2H), 4.27 (q, J = 7.1 Hz, 2H), 3.95 (s, 1H), 3.90 (ddd, J = 11.4, 9.9, 3.1 Hz, 1H), 3.65 (ddt, J = 10.3, 4.4, 2.2 Hz, 1H), ,2.58 – 2.53 (m, 1H), 2.08 – 1.97 (m, 1H), 1.89 (s, 1H), 1.94 – 1.85 (m, 1H), 1.77 – 1.58 (m, 3H), 1.35 (t, J = 7.1 Hz, 3H).


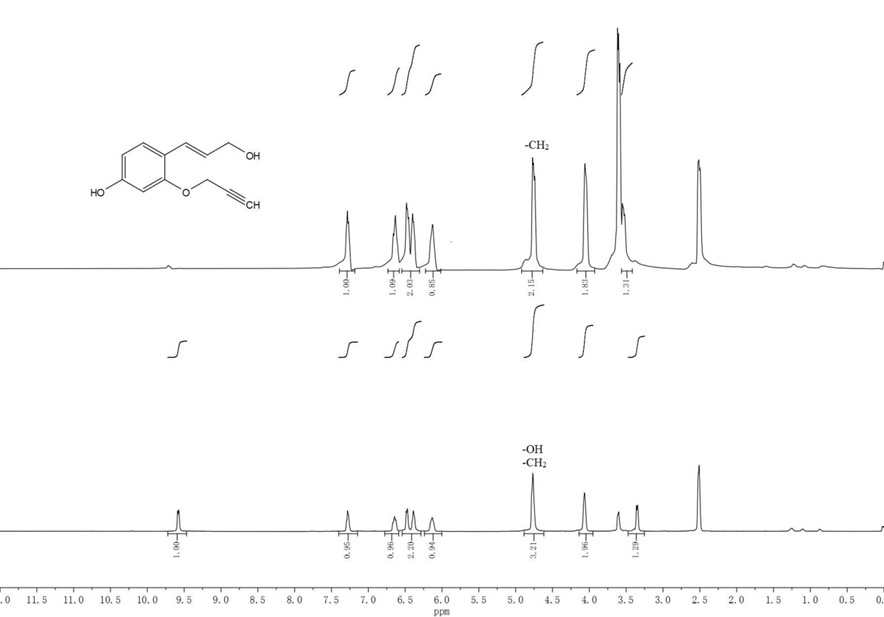


**Supplementary Figure S13.** ^1^H-NMR spectrum of 2-O-propargyl *p*-coumarol alcohol (VI).

Product Ⅴ (1.44g) was put into a three necked flask with 20ml of tetrahydrofuran for under nitrogen. 20ml of DIBAL-H 1.5M in toulene was added dropwise in a low temperature bath at 0 ℃ through a constant pressure funnel within 30min. After stirring and reacting for 4 hours, 2ml of methanol was added to terminate the reaction. 30ml Potassium sodium tartrate and 40ml ethyl acetate were added and stirred at room temperature for 1h to separate the ester layer. The water layer was extracted with 20ml ethyl acetate. The collected ester layer was dried with anhydrous sodium sulfate overnight. After filtration, vacuum evaporation, and recrystallize with n-hexane/ether (2:1, v/v), a white solid was obtained, with a yield of 68% and a melting point of 106.1~108.3 ℃. ^1^H NMR (600 MHz, DMSO-d_6_) δ 9.61 – 9.55 (m, 1H), 7.31 – 7.24 (m, 1H), 6.65 (dd, J = 17.3, 9.7 Hz, 1H), 6.48 (d, J = 10.0 Hz, 1H), 6.38 (d, J = 9.0 Hz, 1H), 6.13 (ddq, J = 12.6, 9.1, 5.2 Hz, 1H), 4.76 (q, J = 10.3, 9.4 Hz, 3H), 4.07 (p, J = 5.7 Hz, 2H), 3.38 – 3.32 (m, 1H).


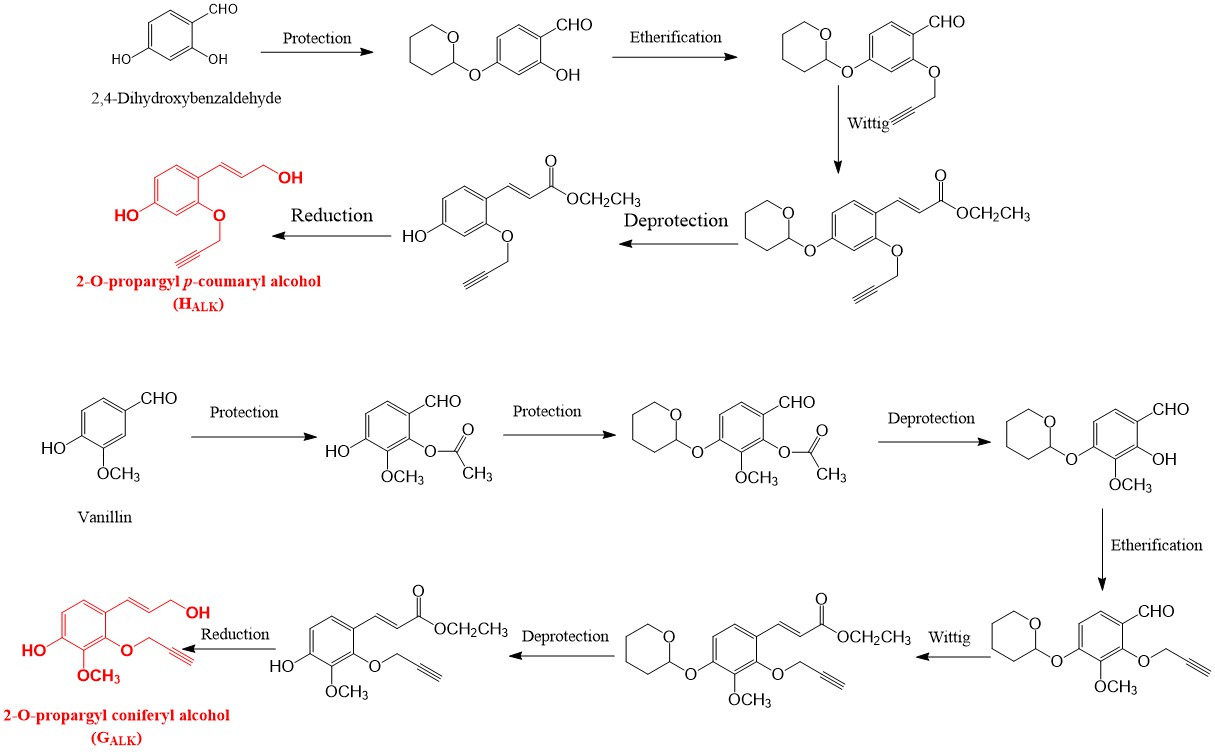


**Supplementary Figure S14.** Preparation of 2-O-propargyl coniferyl alcohol (G_ALK_).


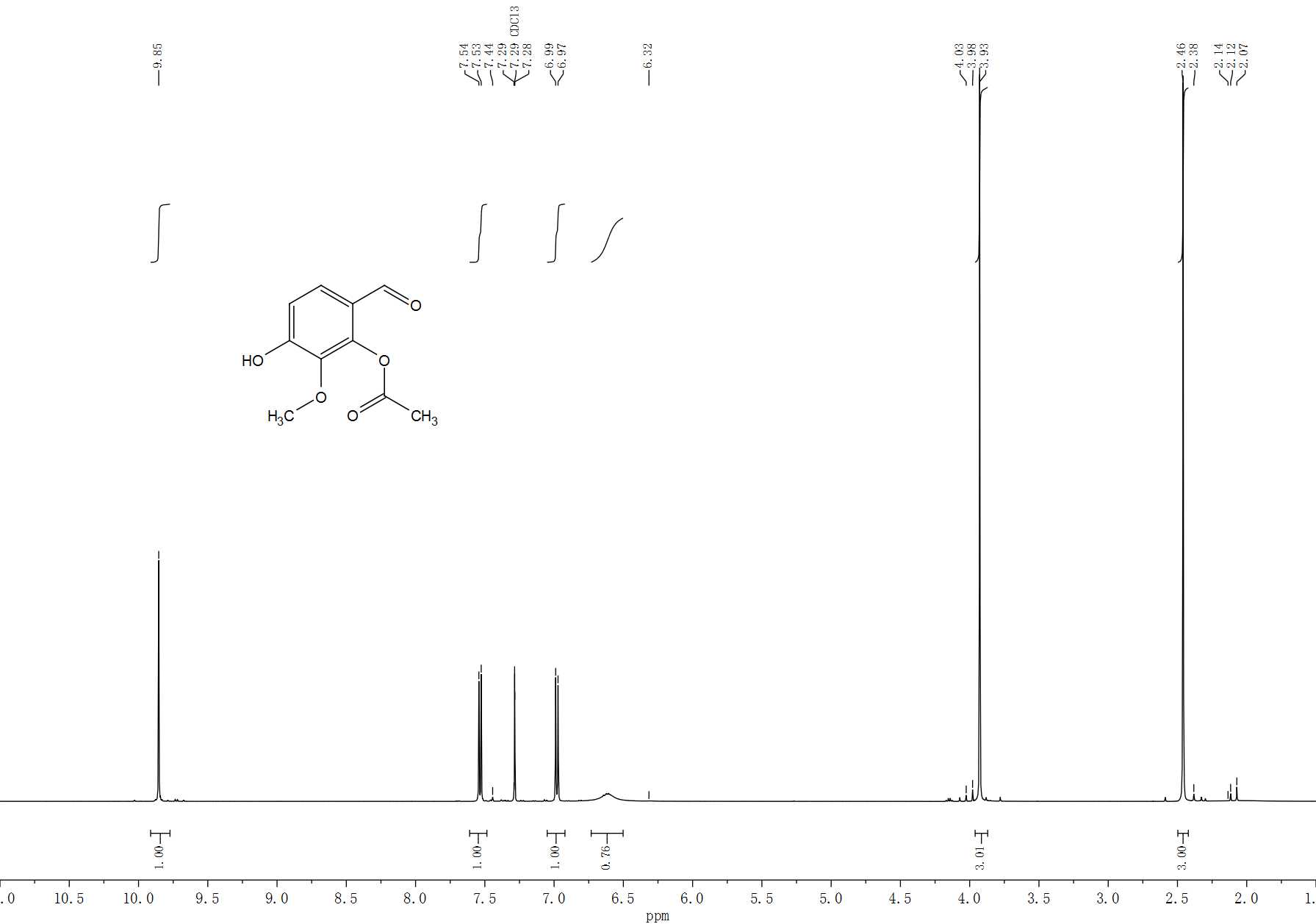


**Supplementary Figure S15.** ^1^H-NMR spectrum of 4-hydroxy-3-methoxy 2-O-acetyl benzaldehyde (Ⅱ).

Vanillin (1g) was added into a bottle filled with 60ml of acetic acid. 2.34g of (Diacetoxyiodo)benzene dissolved in 40ml of acetic acid was added dropwise by constant pressure funnel. As the addition of acetic acid solution of (Diacetoxyiodo)benzene, the color gradually changed from colorless to yellow, and finally to amber transparent liquid. After stirring at room temperature for 3 days, the solution was evaporated under reduced pressure. The target product was separated by column chromatography (n-hexane/ethyl acetate, 7:3, v/v). A light yellow solid powder was obtained after evaporation under reduced pressure (yield of 57.92%). m.p. 106.2~108.7 ℃. ^1^H NMR (500 MHz, Chloroform-d) δ 9.85 (s, 1H), 7.53 (d, J = 8.5 Hz, 1H), 6.98 (d, J = 8.5 Hz, 1H), 6.73 – 6.50 (m, 1H), 3.93 (s, 3H), 2.46 (s, 3H).


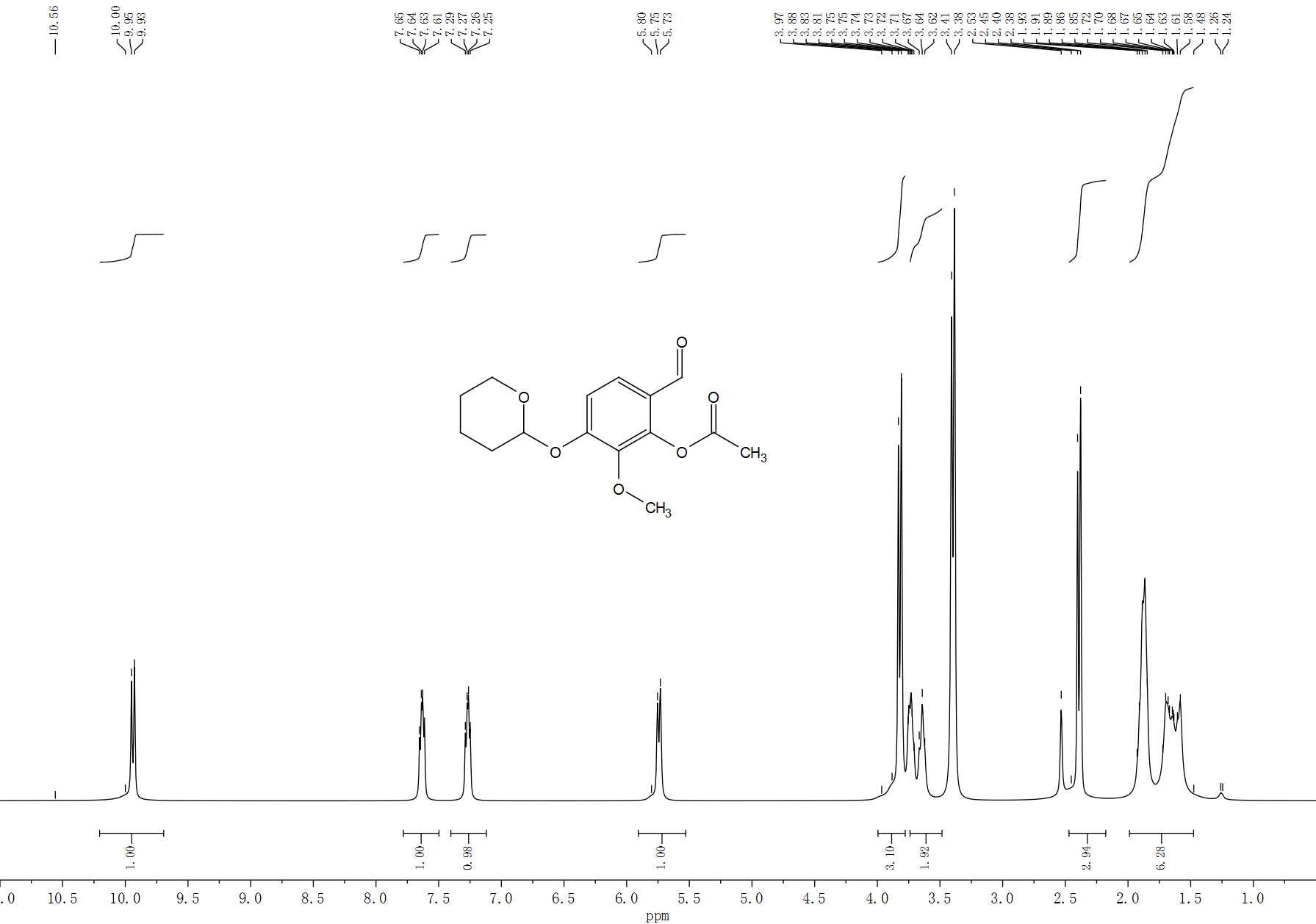


**Supplementary Figure S16.** ^1^H-NMR spectrum of 4-O-tetrahydropyranyl-3-methoxy-2-O-acetyl benzaldehyde(Ⅲ).

Compound II (0.967g) and 2,6-Dimethylpyridinium *p*-Toluenesulfonate (0.088g) were dissolved in a three necked flask with 25mL of dichloromethane and stirred at room temperature. About 1.3ml of 3,4-dihydro-2H-pyran solution was added dropwise by constant pressure funnel. The reaction mixture was heated at 50 ℃ to reflux over 3.5 hours, then 70 mL of ether and 50ml of distilled water were added. The ether layer was collected and washed again with 50ml of distilled water. The collected ether layer was dried over anhydrous sodium sulfate overnight. After filtration and vacuum evaporation, the target product was separated by chromatography (n-hexane/ethyl acetate, 3:1, v/v). White crystal product III was obtained after vacuum evaporation (yield of 65%). m.p. 85.4~87.3 ℃. ^1^H NMR (600 MHz, DMSO-d_6_) δ 9.94 (d, J = 14.4 Hz, 1H), 7.78 – 7.50 (m, 1H), 7.40 – 7.12 (m, 1H), 5.74 (d, J = 13.7 Hz, 1H), 3.82 (d, J = 14.4 Hz, 3H), 3.74 – 3.48 (m, 2H), 2.39 (d, J = 14.4 Hz, 3H), 1.99 – 1.48 (m, 6H).


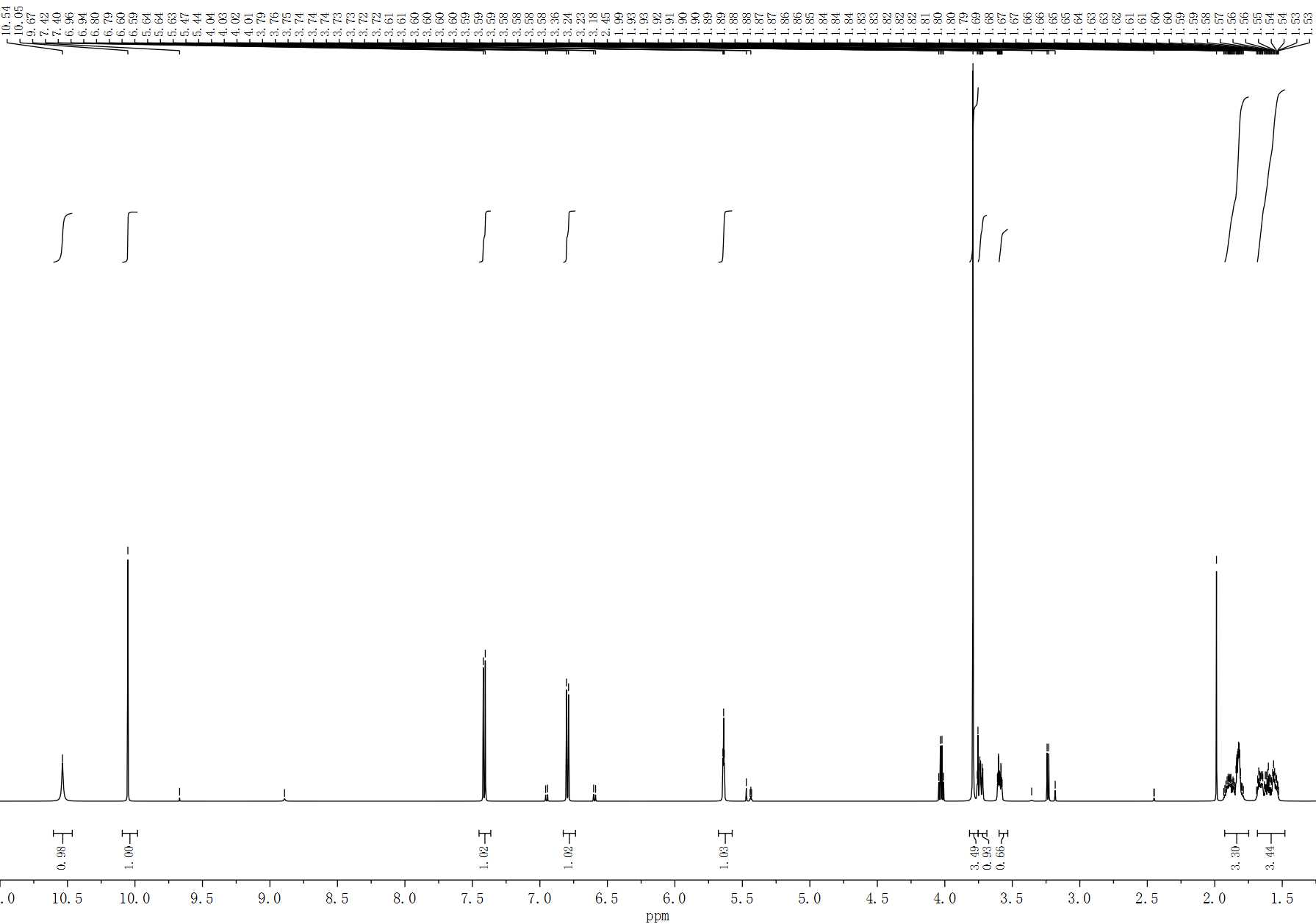


**Supplementary Figure S17.** ^1^H-NMR spectrum of 4-O-tetrahydropyranyl-3-methoxy-2-hydroxy benzaldehyde (IV).

Compound III (1.22g) was dissolved in 10ml of tetrahydrofuran and 30ml of methanol. Then, 0.166g of sodium hydroxide was added and reacted for 5 h at room temperature. Afterwards, 40ml of ethyl acetate and 80ml of distilled water were added. The water layer was separated, which then was extracted by ethyl acetate addition (40 × 2). The ester layer was collected and dried overnight over anhydrous sodium sulfate. A light yellow transparent oily liquid IV was got with a yield of 88% after filtration and vacuum evaporation. ^1^H NMR (600 MHz, DMSO-d_6_) δ 10.54(s, 1H), 10.05 (s, 1H), 7.41 (d, J = 8.8 Hz, 1H), 6.80 (d, J = 8.9 Hz, 1H), 5.64 (t, J = 3.1 Hz, 1H), 3.79 (s, 3H), 3.75 – 3.69 (m, 1H), 3.60 – 3.53 (m, 1H), 1.93 – 1.75 (m, 3H), 1.68 – 1.48 (m, 3H).


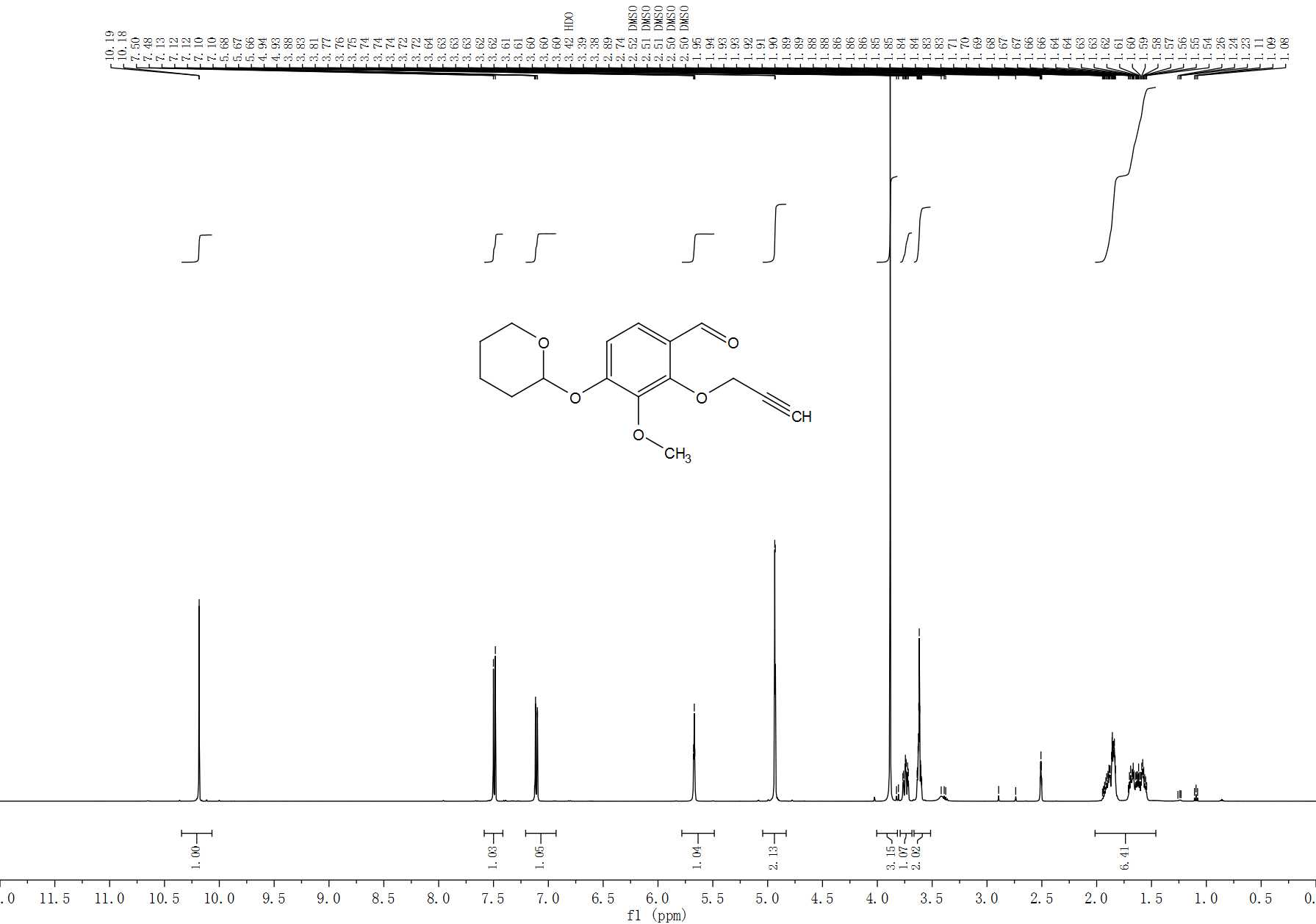


**Supplementary Figure S18.** ^1^H-NMR spectrum of 4-O-tetrahydropyranyl-3-methoxy-2-O-propargyl benzaldehyde (V).

Compound Ⅴ was synthesized by compound Ⅳ. The process was the same as 2-O-PPA (2) (yield of 88.86%). m.p. 56.3～58.2℃. ^1^H NMR (500 MHz, DMSO-d_6_) δ 10.18 (d, J = 0.8 Hz, 1H), 7.49 (d, J = 8.9 Hz, 1H), 7.11 (dd, J = 9.0, 0.8 Hz, 1H), 5.67 (t, J = 2.9 Hz, 1H), 4.93 (d, J = 2.4 Hz, 2H), 3.88 (s, 3H), 3.74 (ddd, J = 11.4, 9.9, 3.4 Hz, 1H), 3.67 – 3.51 (m, 1H), 2.01 – 1.46 (m, 6H).


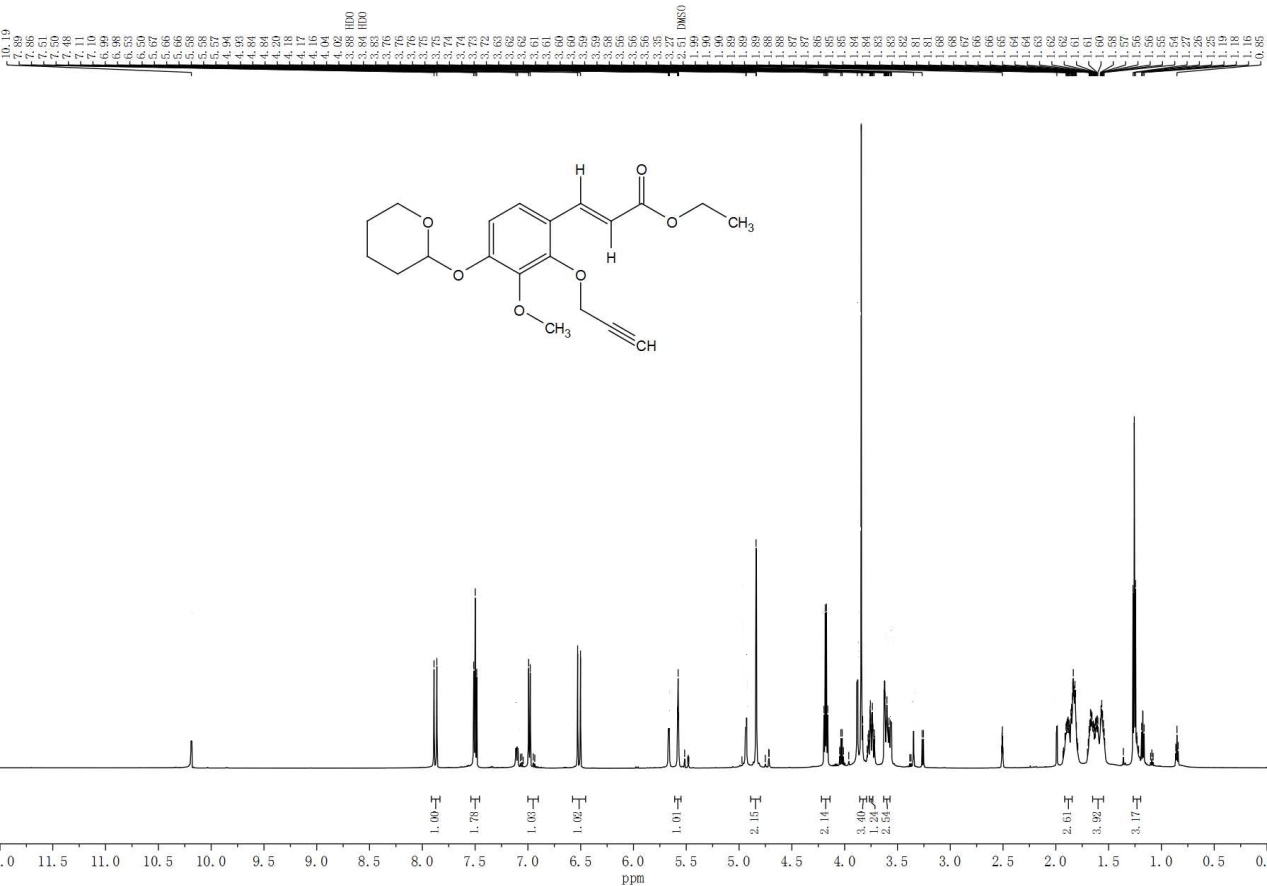


**Supplementary Figure S19.** ^1^H-NMR spectrum of 4-O-tetrahydropyranyl-3-methoxy-2-O-propargyl cinnamate ethyl ester (Ⅵ).

The synthesis method was the same as 2-O-PPA (3) by compound Ⅵ. The product was a white solid, with a yield of 72% and a melting point of 47.1~48.7 ℃. ^1^H NMR (600 MHz, DMSO-d_6_) δ 7.88 (d, J = 16.1 Hz, 1H), 7.50 (t, J = 8.6 Hz, 2H), 6.99 (d, J = 8.9 Hz, 1H), 6.52 (d, J = 16.1 Hz, 1H), 5.58 (t, J = 3.1 Hz, 1H), 4.84 (d, J = 2.5 Hz, 2H), 4.18 (q, J = 7.1 Hz, 2H), 3.83 (s, 3H), 3.76 – 3.73 (m, 1H), 3.63 – 3.57 (m, 2H), 1.92 – 1.85 (m, 3H), 1.60 (dddd, J = 35.1, 17.3, 8.2, 4.2 Hz, 3H), 1.25 (d, J = 7.1 Hz, 3H).


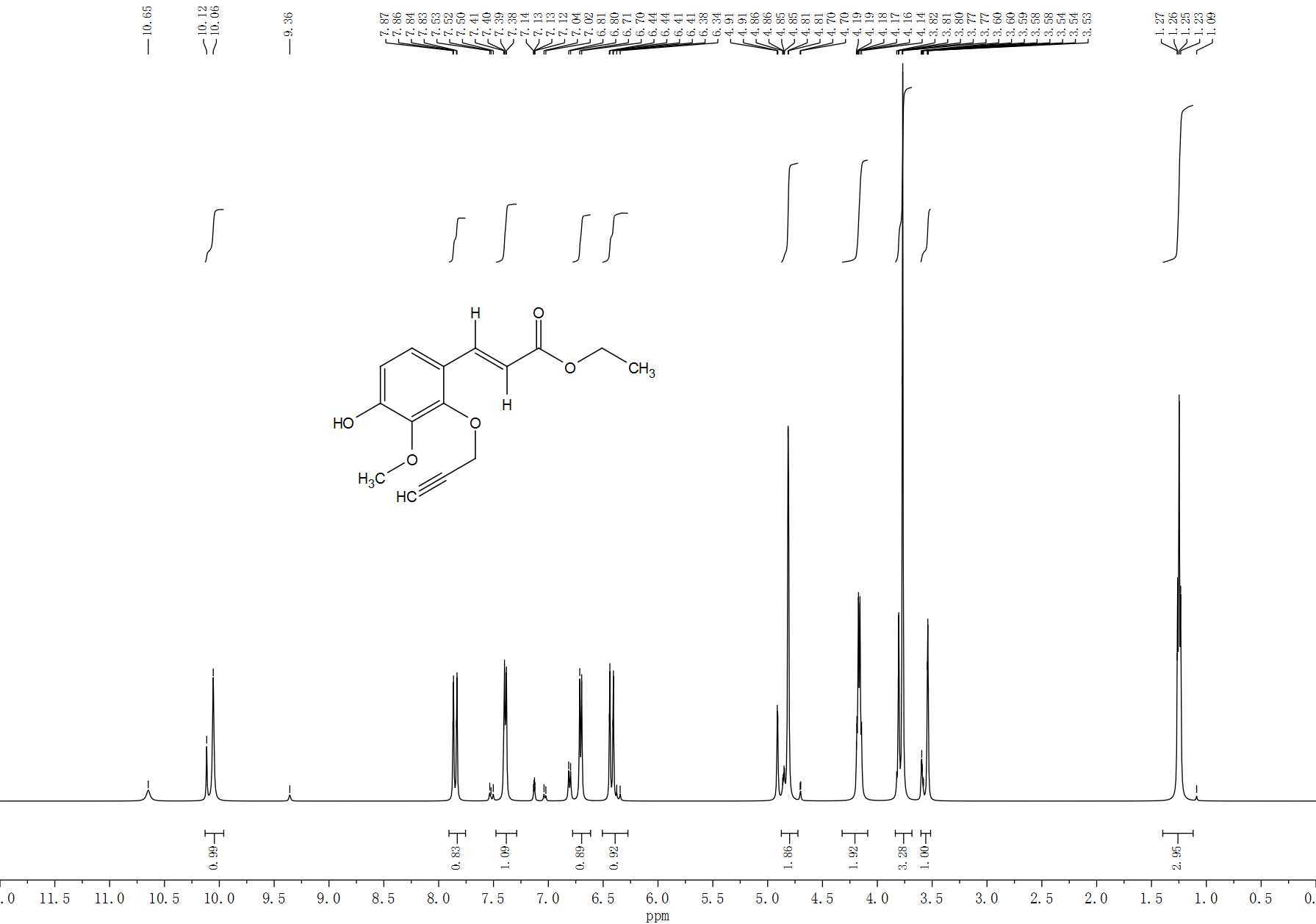


**Supplementary Figure S20.** ^1^H-NMR spectrum of ethyl 4-hydroxy-3-methoxy-2-O-propargyl cinnamate ethyl ester (VII).

Product Ⅵ (1.71g) was put into a pear shaped bottle containing 50ml of tetrahydrofuran solution and 50ml of methanol. After stirring at room temperature for 2 minutes, 0.2ml of 2mol/L HCL was added dropwise, and stirred for another 40 minutes. Then, 40ml of ethyl acetate and 70ml of distilled water were added to extract and separate the solution. The ester layer was collected, which was washed by 70ml of distilled water. After drying over anhydrous sodium sulfate, the ester layer was filtrated and evaporated under reduced pressure at 40 ℃. The target product was separated by chromatography (n-hexane/ethyl acetate, 8:1). White product VII was obtained after vacuum evaporation with a yield of 64% and a melting point of 76.7-78.9 ℃. ^1^H NMR (500 MHz, DMSO-d_6_) δ 10.09 (d, J = 29.8 Hz, 1H), 7.85 (dd, J = 16.1, 2.4 Hz, 1H), 7.39 (dd, J = 8.7, 3.7 Hz, 1H), 6.70 (d, J = 8.7 Hz, 1H), 6.42 (dd, J = 16.1, 2.3 Hz, 1H), 4.81 (d, J = 2.6 Hz, 2H), 4.32 – 4.09 (m, 2H), 3.84 – 3.68 (m, 3H), 3.57 (dt, J = 27.8, 2.5 Hz, 1H), 1.25 (t, J = 7.0 Hz, 3H).


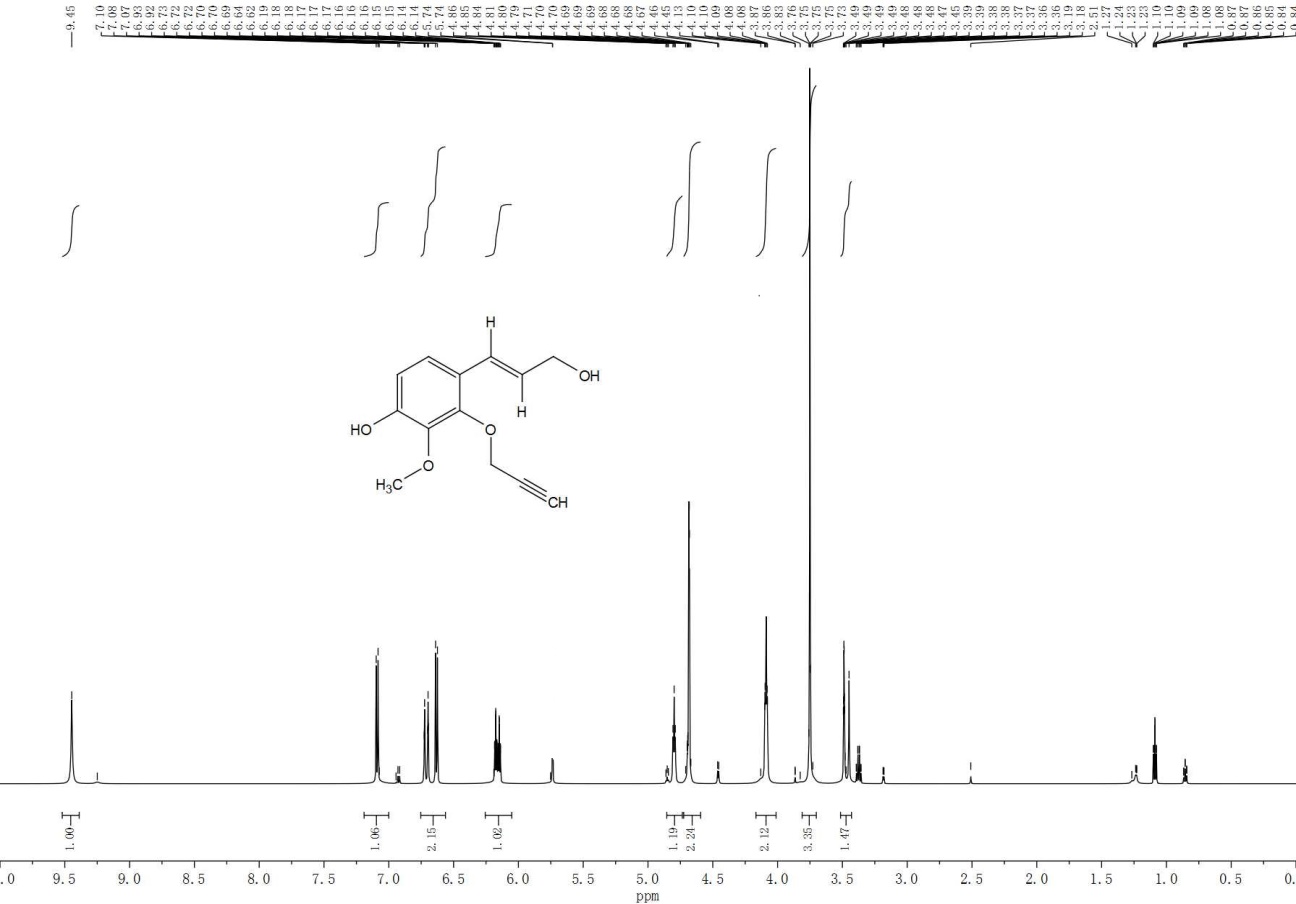


**Supplementary Figure S21.** ^1^H-NMR spectrum of 2-O-propargyl coniferyl alcohol (VIII).

The synthesis method was the same as 2-O-PPA (5) by compound Ⅷ. The product was a light yellow oily liquid, with yield of 65%. ^1^H NMR (600 MHz, DMSO-d_6_) δ 9.45 (s, 1H), 7.09 (d, J = 8.6 Hz, 1H), 6.75 – 6.56 (m, 2H), 6.16 (dtd, J = 16.0, 5.4, 0.9 Hz, 1H), 4.80 (t, J = 5.5 Hz, 1H), 4.72 – 4.59 (m, 2H), 4.17 – 4.01 (m, 2H), 3.75 (d, J = 1.0 Hz, 3H), 3.51 – 3.43 (m, 1H).
